# The impact of different types of exercise on sleep in sedentary populations: a systematic review and network meta-analysis

**DOI:** 10.7717/peerj.21037

**Published:** 2026-06-09

**Authors:** Jia Guo, Jiakai Tang, Junpeng Jiang, Jianyu Li, Ruihan Zhu, Guochun Liu, Chunmei Cao

**Affiliations:** Division of Sports Science and Physical Education, Tsinghua University, Beijing, Haidian, China

**Keywords:** Exercise, Sedentary population, Sleep, Network meta-analysis

## Abstract

**Background:**

The widespread prevalence of sleep disturbances poses substantial challenges to both public health and individual well-being. Exercise may serve as an effective non-pharmacological intervention. However, the comparative efficacy of different exercise modalities and the influence of participant or intervention characteristics on treatment response remain unclear.

**Methods:**

We conducted a systematic review and network meta-analysis of randomized controlled trials (RCTs) identified from PubMed, Web of Science, MEDLINE, SPORTDiscus, EBSCO, and Scopus (inception to November 2024). A total of 31 RCTs (*n* = 1,420) evaluating aerobic exercise (AE), resistance training (RT), high-intensity interval training (HIIT), mind–body exercise (ME), prolonged time of exercise (PTE), and mixed modalities (MIX) on sleep outcomes in sedentary adults with insomnia were included. Study quality was assessed using the Jadad scale, and analyses were performed in Stata 17.0 using a random-effects model.

**Results:**

This study aimed to explore the effects of different types of exercise on sleep outcomes (sleep questionnaire score (SCORE), sleep latency (SL), sleep efficiency (SE), total sleep time (TST), sleep quality (SQ), and wake time after sleep onset (WASO)). The meta-analysis showed that exercise significantly improved SCORE (Hedges’ g = 1.00, 95% CI [0.59–1.41]). Subgroup analyses indicated that the largest improvements occurred in individuals aged 45–59 (Hedges’ g = 1.97, 95% CI [0.10–3.84]), those classified as overweight (Hedges’ g = 0.27, 95% CI [0.04–0.51], and in interventions lasting 0–8 weeks (Hedges’ g = 7.18, 95% CI [5.87–8.48]) or 8–16 weeks (Hedges’ g = 0.73, 95% CI [0.32–1.13]). A significant effect was also observed in female-only samples (Hedges’ g = 0.95, 95% CI [0.04–1.87]). Network meta-analysis further suggested that: (1) PTE improved SCORE, SE, TST, WASO, and SQ; (2) AE enhanced SE, TST, and SQ; (3) both MIX and ME had positive effects on SE and TST.

**Conclusion:**

Different exercise modalities show distinct benefits for specific sleep outcomes in sedentary adults with insomnia: PTE improved multiple domains (SCORE, SE, TST, WASO, SQ), AE enhanced SE, TST, and SQ, and MIX and mind–ME benefited SE and TST. Programs lasting 8–16 weeks yielded the most consistent effects. These findings support tailored exercise selection, though results should be interpreted cautiously due to limited evidence for some modalities.

## Introduction

Healthy sleep is crucial for optimal physical and mental functioning, contributing to physiological restoration, cognitive performance, and emotional regulation (Davidson & Marcusson-Clavertz, 2023; Slavish et al., 2024). Individuals suffering from sleep problems often experience profound social and economic burdens, including reduced work productivity, increased healthcare costs, and impaired quality of life (Davidson & Marcusson-Clavertz, 2023; Mello et al., 2007; Mystakidou et al., 2007). Moreover, chronic sleep problems, such as insomnia, have been associated with elevated risks of various comorbidities, such as cardiovascular diseases, diabetes, depression, and anxiety disorders (Nadarajah et al., 2025; Tobaldini et al., 2019). Alarmingly, epidemiological studies suggest that 30% to 50% of the global population may be affected by insomnia symptoms at some point in their lives (Ford et al., 2014), highlighting its widespread prevalence and public health significance.

The modern era, marked by rapid technological advancements, has undeniably enhanced convenience in daily life. Nevertheless, technological convenience has unintentionally encouraged sedentary lifestyles characterized by prolonged sitting (Shrestha et al., 2016). Sedentary behavior affects sleep both directly, by disrupting circadian rhythms and reducing physical activity, and indirectly through increased systemic inflammation and psychological stress (Manifield et al., 2024; You et al., 2023). This negatively impacts sleep quality (SQ), worsening overall health and creating a declining physical and mental well-being cycle. These findings highlight the need to decrease sedentary behavior and increase the level of physical activity as a key public health issue, with implications for improving sleep and general health (Hasan et al., 2022; Manifield et al., 2024; You et al., 2023).

Given the adverse effects of prolonged sedentary behavior, structured physical exercise emerges as a promising non-pharmacological strategy (Benca, 2005; Riedel et al., 2024). Long-term aerobic exercise (AE) has been shown to significantly improve both objective and subjective SQ in older sedentary individuals, as well as reduce circulating pro-inflammatory markers (King et al., 1997; Reid et al., 2010). Additionally, mind-body exercises (ME) such as tai chi, Pilates, and yoga are also promising options for enhancing SQ (Boing et al., 2023; García-Soidán et al., 2014; Hasan et al., 2022; Innes & Selfe, 2012). RT is crucial for older adults as it helps strengthen muscles, improve dynamic balance, and enhance SQ (Baker et al., 2021). To compare different exercise modalities, Al-Jiffri & Abd El-Kader (2021) conducted a study comparing the effects of 6 months of AE *vs*. RT on sleep parameters and inflammatory markers. Their findings indicated that AE was more effective and appropriate for sedentary individuals with insomnia. A recent meta-analysis by Riedel et al. (2024) further confirmed that exercise significantly improves sleep in individuals with insomnia (SMD = 0.90 for subjective outcomes). However, this and other existing reviews do not differentiate between specific exercise modalities or provide a comparative effectiveness ranking—particularly in sedentary adults.

Nevertheless, uncertainty remains about which exercise modality is most effective for improving sleep in sedentary individuals. This study therefore sought to determine which exercise modality best improves sleep in sedentary individuals through a systematic review and network meta-analysis.

## Methods

### Registration

This systematic review and NMA follow the PRISMA guidelines and Cochrane Handbook methodologies. The protocol is registered in PROSPERO (CRD42024611291). The PRISMA-NMA checklist is provided in Table S1.

### Search strategy

The literature was searched from the following databases: PubMed, Web of Science, Medline, SPORT Discus, EBSCO, and SCOPUS. The search covered the period from inception to November 2024, with only English-language articles included. The search terms used in this review included: *sedentary, inactive, desk-bound, sleep, sleep quality, sleep onset latency, total sleep time, fragmentation index, insomnia, wake after sleep onset, sleep efficiency, exercise, physical activity, training, swimming, yoga, walking, aerobic, climbing, running, cycling, bicycling, resistance training, endurance training, Pilates*, and *randomized controlled trial*. The details of the search query are provided in Table S2.

### Eligibility criteria

The inclusion criteria for the literature adhered to the PICOS framework. The search strategy was designed based on the PICOS principle: (P) Population: individuals with sedentary lifestyles; (I) Intervention: acute or long-term exercise programs (Table 1); (C) Comparator: absence of exercise; (O) Outcomes: Outcome measures included sleep questionnaire score (SCORE), sleep latency (SL), sleep efficiency (SE), total sleep time (TST), sleep quality (SQ) and wake time after sleep onset (WASO), measured using subjective assessment tools (*e.g*., Pittsburgh Sleep Quality Index, PSQI) or objective sleep measurement tools (*e.g*., Polysomnography, PSG). At least one of these indicators must be included in the primary outcomes. (S) Study design: only randomized controlled trials (RCT) are considered for inclusion. Studies were excluded if any of the following conditions applied: (1) The intervention did not involve exercise modalities, or there was no difference in exercise methods between groups; (2) the article was a duplicate publication or was not written in English.

**Table 1:** The classifications of exercise training.

Exercise type	Definition
**Aerobic Exercise (AE)**	Physical activities primarily powered by aerobic energy metabolism, such as brisk walking, jogging, aerobics, cycling, and swimming (Al-Jiffri & Abd El-Kader, 2021; Cardoso et al., 2010; Reid et al., 2010).
**Resistance Training (RT)**	Exercises aimed at enhancing muscle strength or endurance by overcoming resistance, including body weight or external forces (Al-Jiffri & Abd El-Kader, 2021; Baker et al., 2021; Cardoso et al., 2010).
**High-Intensity Interval Training (HIIT)**	A training method involving repeated high-intensity exercise bouts interspersed with recovery intervals (Frimpong et al., 2021; Oliveira et al., 2022).
**Mind-Body Exercise (ME)**	Activities that integrate mental focus, physical movement, and behavioral awareness, such as yoga, qigong, Baduanjin, Tai Chi, and relaxation techniques (Frye et al., 2007; Irwin et al., 2014; Li et al., 2020).
**Prolonged Time of Exercise (PTE)**	Interventions that increase participants’ overall physical activity levels without specifying a particular exercise modality (Quist et al., 2019; Wang & Boros, 2021; Yeung et al., 2018).
**Mixed Exercise (MIX)**	Combinations of two or more of the above exercise types (Hortobágyi et al., 2021; Rayward et al., 2020).
**Control Training (CT)**	Do not receive any exercise intervention.

### Data extraction

After database screening, all articles were imported into Zotero software (version 6.0.30) to remove duplicates. Subsequently, two researchers (Jia Guo and Jiakai Tang) independently screened the titles and abstracts, and a third researcher (Jianyu Li) resolved any discrepancies.

Data extraction included the following: (1) Basic study information (first author, publication year, *etc*.); (2) participant demographics; (3) intervention details (type of exercise, applied load, frequency per week, duration per session in minutes, total weeks); (4) outcome assessment methods and indicators; (5) quality assessment results of the literature.

### Quality assessment

The Jadad scale (maximum score = 7) was used to assess study quality (Guo et al., 2024; Pan et al., 2024). It remains widely adopted in exercise trials due to the practical challenges of blinding participants and outcome assessors. By focusing on randomization, blinding, and handling of withdrawals, the Jadad scale offers a pragmatic and contextually appropriate approach to quality appraisal in non-pharmacological trials. The evaluation focused on four aspects: (1) Correct application of randomization methods; (2) adequate concealment of randomization; (3) proper implementation of blinding; (4) description of participant withdrawal or dropout. Items 1–3 were rated as 0, 1, or 2, while item 4 was rated as 0 or 1. Studies scoring 1–3 were classified as low quality, whereas those scoring 4–7 were classified as high quality.

### Statistical analysis

The statistical analysis was conducted using RevMan 5.4 software for pairwise meta-analysis and the random-effects model within the frequentist framework of Stata 17.0 for NMA (network, mvmeta, metan and metareg packages). The effect size was estimated using the difference in changes before and after the intervention between the experimental and control groups. The change’s standard deviation (SD) for continuous variables was calculated using the following formula:

$S{D_{change}} = \sqrt {SD_{baseline}^{\matrix{ 2 \cr } } + SD_{final}^2 - \left( {2 \times Corr \times S{D_{baseline}} \times S{D_{final}}} \right).}$

The correlation coefficient (Corr) was set to 0.5. Because sleep outcomes were assessed using different scales, we used Hedges’ g as the effect size metric, along with its 95% confidence interval. Network plots were generated using the network package in Stata. If closed loops existed among studies, the node-splitting method was applied to test consistency. In cases of inconsistency, the inconsistency model was used. Rankings of interventions were determined by calculating the surface under the cumulative ranking curve (SUCRA), where a higher SUCRA value indicated better intervention effectiveness. Bias was assessed using funnel plots. Following the Cochrane Handbook guidelines, reverse scaling was applied to certain outcomes to ensure consistency across results when effect measures contradicted the primary direction.

## Result

### Literature selection

**Figure 1:**
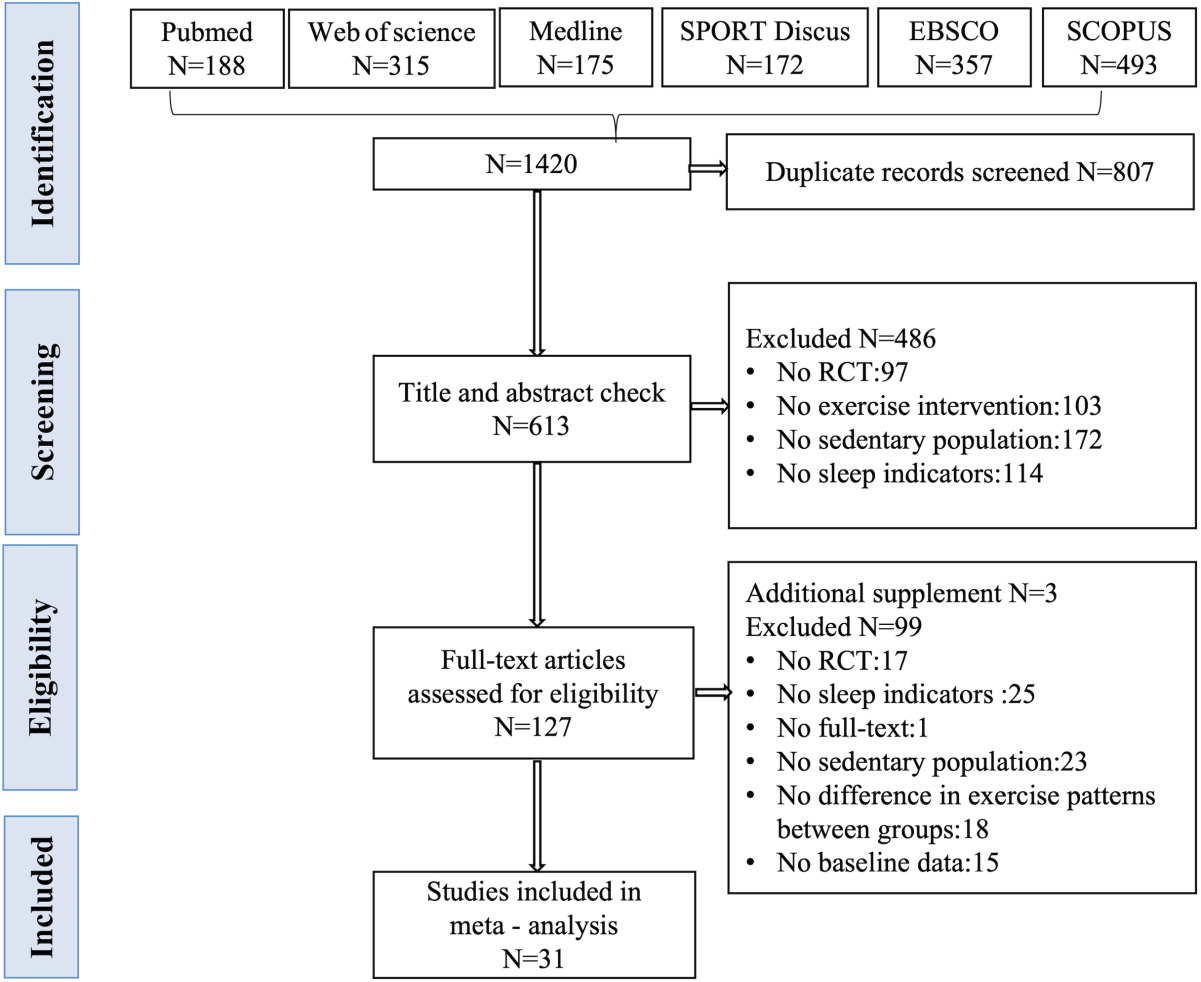
The flowchart of systematic review and meta-analysis (PRISMA) depicts the study selection process.

The process of selecting studies for inclusion is illustrated in Fig. 1. A total of 1,420 articles meeting the specified criteria were identified through database searches. After removing 807 duplicate records, 613 articles remained. Screening of titles and abstracts led to the exclusion of 486 articles, and a subsequent full-text review resulted in the removal of an additional 99 articles. Three additional studies were identified through reference list screening, resulting in a final total of 31 articles included in the analysis.

### Characteristics of the included studies

Table 2 summarizes the characteristics of the included studies, published between 1997 and 2024 across ten countries. These studies were published between 1997 and 2024 and conducted in the USA (*n* = 14), Australia (*n* = 3), Brazil (*n* = 3), the UK (*n* = 2), Spain (*n* = 2), Saudi Arabia (*n* = 2), China (*n* = 1), Denmark (*n* = 1), Hungary (*n* = 1), and Japan (*n* = 1). Among them, three studies were three-arm trials, while the remaining 28 were two-arm trials. A total of 1,984 sedentary participants were included (1,121 in the intervention groups and 913 in the control groups), comprising 661 males and 1,138 females (seven studies included only males or females, and four studies did not report participant gender). The participants’ ages ranged from 20.1 to 72.3 years, with BMIs between 21.69 and 33.74, encompassing young, middle-aged, and older adult populations.

**Table 2:** Characteristics of included studies.

	Basic information of two-arm studies												
	Study	Region	Sample size (n)	Sex (M/F)	Age (years)	BMI	Intervention	Intensity/Load	Frequency (times/week)	Duration (minutes/session)	Intervention period (weeks)	Sleep assessment method	Sleep outcomes
1	King et al. (1997)	USA	43 (T:20, C:23)	M = 14, F = 29	61.4 (7.06)	–	AE (brisk walking)	Moderate-intensity (60% to 75% of heart rate reserve)	4	30–40	16	PSQI	SCORE, SL, SE, TST, SQ
2	King et al. (2002)	USA	85 (T:45, T:40)	M = 100	62.7 (9.1)	–	AE (brisk walking)	Moderate-intensity (60% to 75% of heart rate reserve)	4	30–40	12	PSQI	SL, TST, SQ
3	de Jong et al. (2006)	USA	181 (T:79, C:102)	M = 80, F = 101	59.1 (2.6)	26.8 (3.4)	AE	Moderate-intensity	3	60	24	TAAQOL	SCORE
4	Frye et al. (2007)	USA	72 (ME:31, AE + RT:30, C:23)	M = 30, F = 54	69.2 (9.26)	28.96 (4.31)	Taichi AE + RT	– Low to moderate-intensity	5 6	60 61	12 12	PSQI	SCORE
5	King et al. (2008)	USA	66 (T:36, C = 30)	M = 22, F = 44	61.42 (6.7)	27.55 (3.98)	AE	Moderate-intensity (60% to 85% of peak heart rate)	5	60	12	PSG, PSQI	SCORE, SL, SE, TST
6	Reid et al. (2010)	USA	17 (T:10, C:7)	M = 1, F = 16	62.6 (4.3)	26.5 (4.6)	AE	Moderate-intensity (60% to 75% of peak heart rate)	3	40	16	PSQI	SCORE, TST, SL, SE, SQ
7	Innes & Selfe, 2012	USA	75 (T:38, C:37)	F = 75	58.65 (15)	–	ME (Yoga)	–	2	90	8	PSQI	SCROE, TST, SL, SE, SQ
8	Kline et al. (2012)	USA	43 (T1:27, C:16)	M = 24, F = 19	47.0 (1.86)	>25	AE + RT	Moderate-intensity	6	150	12	FOSQ-10, 10-item Functional Outcomes of Sleep Questionnaire;	SCORE
							ME (Stretching)	–	2	60	12		
9	Oudegeest-Sander et al. (2012)	UK	21 (T:11, C:10)	M = 11;F = 10	69 (3)	25.1 (2.5)	AE	Moderate-intensity (70% to 85% of heart rate reserve)	3	45	24	Accelerometer	SL, TST, SE, WASO
10	García-Soidán et al. (2014)	–	99 (T:51, C:48)	M = 50;F = 49	47.6 (0.8)	24.7 (0.36)	ME (Pilates)	–	2	60	12	Accelerometer, PSQI	SCROE, TST, SL
11	Sternfeld et al. (2014)	USA	248 (T:106, C:142)	F = 248	54.88 (3.62)	26.8 (4.3)	AE	Moderate-intensity (50% to 70% of heart rate reserve)	3	40–60	12	PSQI	SCORE
12	Hartescu, Morgan & Stevinson (2015)	Spain	41 (T:20; C:21)	M = 30;F = 11	59.8 (9.46)	26.29 (4.18)	AE (brisk walking)	Moderate to vigorous intensity	5	30	24	Insomnia Severity Index (ISI)	SCORE
13	Hurdiel et al. (2017)	USA	19 (T:10;C;9)	F = 19	20.1 (1.7)	23 (3.8)	AE	Moderate-intensity (55% to 70% of maximum heart rate)	2	75	12	PSQI, accelerometer	SCORE, TST
14	Yeung et al. (2018)	China	37 (T:18, C:19)	M = 3;F = 34	49.9 (13.6)	22.1 (2.8)	PTE	–	7	30–60	8	ISI, accelerometer	SCORE, SL, TST, SE, WASO
15	El-Kader & Al-Jiffri (2019)	Saudi Arabia	50 (T:25, C:25)	–	65.35 (3.98)	–	AE	Moderate-intensity (60% to 80% of maximum heart rate)	3	40	24	PSG	TST, SE, WASOSQ
16	Quist et al. (2019)	Denmark	72 (AE:22, PTE:24, C:16)	M = 40;F = 32	34 (7)	29.6 (2.6)	AE PTE	Vigorous-intensity (70% VO2peak-reserve) –	5 5	36 (7) 45 (10)	24 24	Accelerometer, PSQI	SCORE, TST
17	Jurado-Fasoli et al. (2020)	Spain	50 (AE + RT:17, HIIT:18, C:15)	23/27	53.4 (2.0)	26.8 (3.8)	AE + RT HIIT	AE: moderate-Intensity (60% to 65% of heart rate reserve); RT: 40–50% of 1RM –	3 3	70 70	12 12	Accelerometer, PSQI	SCORE, TST, SE, WASO
18	Rayward et al. (2020)	Australia	165 (T:110, C:55)	29/136	52 (6.9)	28.42(4.2)	MIX (ME + PTE + RT)	–	7	–	12	PSQI	SCORE
19	Wang & Boros (2020)	Hungary	26 (T:14;C:12)	7/19	24.96 (5.13)	21.69 (2.64)	PTE	–	7	–	4	PSQI	SQ, SL, TST, SE
20	Al-Jiffri & Abd El-Kader, 2021	Saudi Arabia	60 (AE:30, RT:30)	38/22	42.58 (4.22)	33.74 (3.67)	AE	Moderate-intensity (60% to 80% of maximum heart rate)	3	40	24	PSG	SL, TST, SE, WASO
							RT	60–80% 1RM	3	40	24		
21	Baker et al. (2021)	USA	29 (T:15, C:14)	–	68.2 (6.7)	31.2 (8.4)	RT	–	2	60	8	PSQI	SCORE
22	Hortobágyi et al. (2021)	USA	107 (T:53, C:54)	–	60.65 (2.82)	24.75 (2.73)	MIX (ME + AE + RT)	–	3	60	8	PSQI	SCORE
23	Seol et al. (2021) (randomized crossover study)	Japan	10 (T:10, C:10)	F = 20	72.3 (2.3)	21.8 (2.9)	AE	Low-intensity	–	–	acute	PSG	SL, TST, SE, WASO
24	Teychenne et al. (2021)	UK	62 (T:32, C:30)	F = 62	33.31 (3.68)	–	PTE	–	–	–	12	PSQI	SCORE
25	Durante et al. (2022)	Brazil	38 (T:20, C:18)	20/18	52 (7)	30 ± 4	MIX (AE + RT)	Vigorous-intensity	3	50–60	11.65 ± 0.86	PSG	TST
26	McDonough et al. (2022)	USA	64 (T:32, C:32)	20/48	22.8 (3.4)	23.1 (2.6)	MIX (AE + RT)	–	–	6.3 (3.9)	12	Accelerometer	TST,SE
27	Barbosa et al. (2023)	Brazil	40 (T:30, C:20)	–	67 (5)	–	AE	–	3	30	12	Fantastic Life Questionnaire	SCORE
28	Boing et al. (2023)	Brazil	49 (T:25, C:24)	F = 49	55.52 (10.76)	–	ME (Pilates)	–	3	60	12	PSQI	SCORE
29	Brooker et al. (2023)	Australia	57 (T:39, C:18)	14/43	40.1 (14.9)	30.5 (4.14)	AE	Moderate to vigorous intensity	5	50	12	MARCA	TST
30	Lohman et al. (2023)	USA	30 (T:15, C:15)	10/20	50.11 (7.7)	30.34 (5.14)	HIIT	Moderate to vigorous intensity (77–93% of maximum heart rate)	3	23	4	PSQI	SCORE
31	Gale et al. (2024)(randomized crossover study)	Australia	28 (T:28, C:28)	20/8	25.6 ± 5.6	29.5 ± 6.7	PTE	–	–	–	acute	Accelerometer	TST, SE, WASO

**Note:**

AE, Aerobic Exercise; RT, Resistance Training; HIIT, High-Intensity Interval Training; ME, Mind-Body Exercise; PTE, Prolonged Time of Exercise; MIX, Mixed Exercise; CG, Control Group; SCORE, sleep questionnaire score; SL, sleep latency; SE, sleep efficiency; TST, total sleep time; SQ, sleep quality; WASO, wake time after sleep onset.

The exercise modalities included AE, RT, HIIT, ME, PTE, and MIX. The average duration of exercise interventions across all studies was 13.9 weeks, with two studies being single-session experiments. Sleep outcomes were assessed using both subjective and objective tools, including PSQI, PSG, accelerometers, ISI, TAAQOL, FOSQ-10, the Fantastic Life Questionnaire, and MARCA, measuring metrics such as SCORE, SL, SE, TST, SQ, WASO.

### Results of quality assessment

Based on the Jadad scale assessment (Table S3). Overall methodological quality was moderate to high, with fifteen studies scoring ≥4 on the Jadad scale. Adequate randomization methods were described in 21 studies (scoring two points for this criterion), and all studies appropriately addressed participant dropouts. However, only 13 studies implemented adequate allocation concealment, and 18 studies didn’t report the application. Funnel plots were used to assess publication bias for the outcomes SCORE, SE, WASO, TST, and SQ. The results showed symmetrical funnel plots (Fig. S1) for SCORE, TST, and SQ, indicating a low risk of publication bias. However, slight asymmetry was observed in the funnel plots for SL and SE, suggesting a potential small-study effect. Notably, the funnel plot for WASO exhibited significant asymmetry, particularly given the limited number of studies, indicating a risk of publication bias or insufficient sample sizes, which may influence the reliability of the findings.

#### Primary outcome

**Figure 2:**
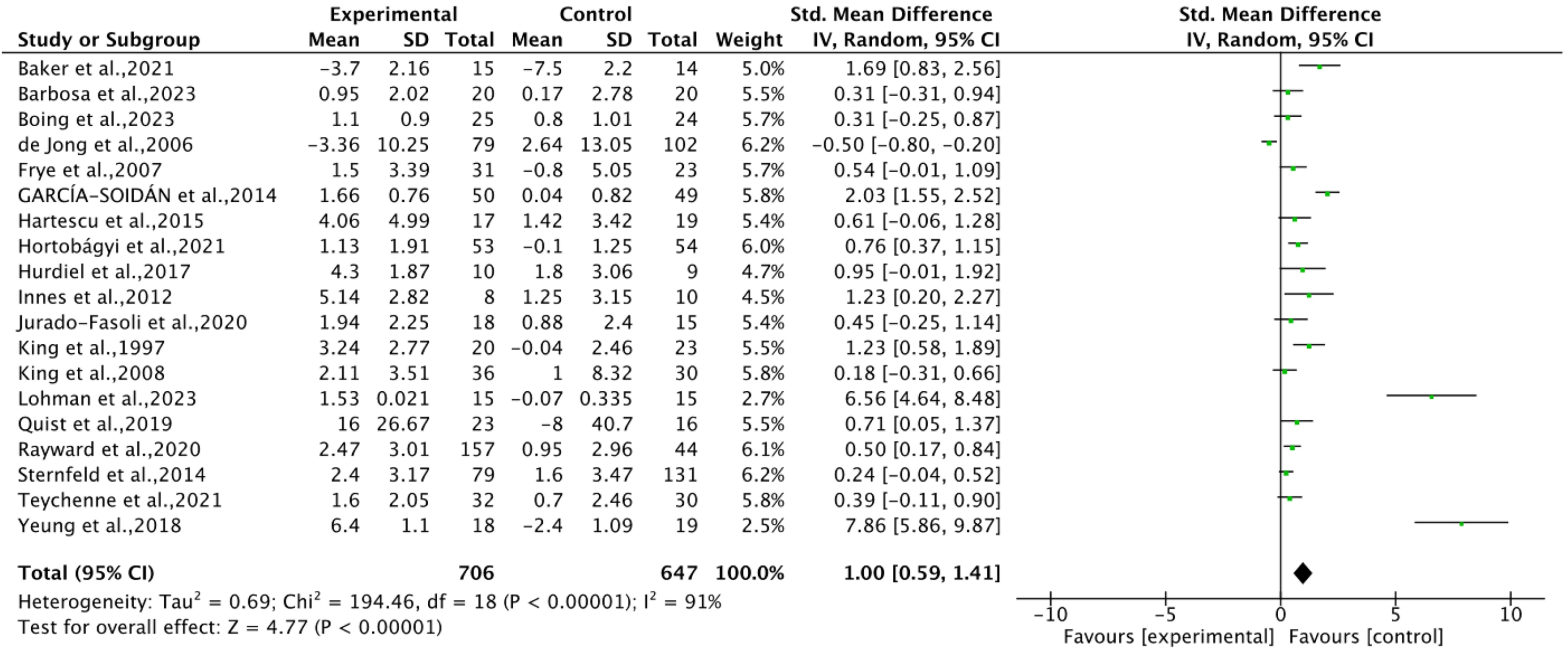
Forest plot of the effects of exercise on SCORE.

The initial meta-analysis evaluated the overall effect of exercise on sleep quality, showing a significant improvement in total SCORE compared with controls. Forest plots displaying individual study results are presented in Fig. 2. The analysis showed that exercise interventions significantly improved subjective sleep scores compared to control conditions (Hedges’ g = 1.00, 95% CI [0.59–1.41]; *p* < 0.001). However, substantial heterogeneity was observed.

#### Secondary outcome

**Figure 3:**
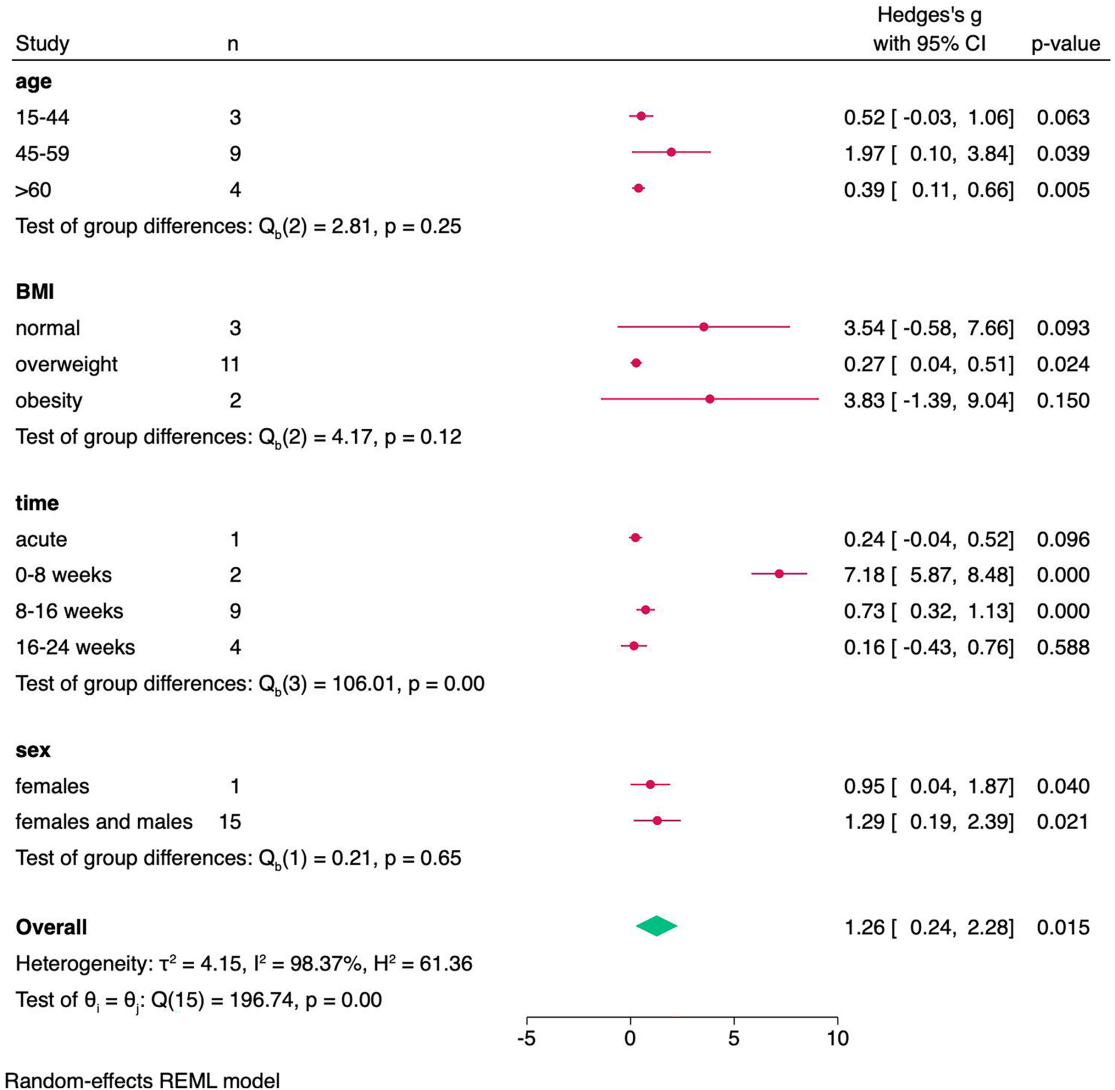
Subgroup analysis results for SCORE.

To explore potential sources of heterogeneity observed in the primary analysis (I² = 98.37%), subgroup analyses (Fig. 3) were conducted based on participant characteristics (age, BMI) and intervention features (duration, sex). Statistically significant between-group differences were not observed for age or BMI categories. However, within-subgroup findings revealed that the largest effect size occurred in adults aged 45–59 years (Hedges’ g = 1.97, 95% CI [0.10–3.84], *p* = 0.039), and among participants classified as overweight (Hedges’ g = 0.27, 95% CI [0.04–0.51], *p* = 0.024). In contrast, intervention duration significantly moderated the effect (Q_b_(3) = 106.01, *p* < 0.001). Programs lasting 0–8 weeks showed the largest effect (Hedges’ g = 7.18, 95% CI [5.87–8.48], *p* < 0.001), followed by those lasting 8–16 weeks (Hedges’ g = 0.73, 95% CI [0.32–1.13], *p* < 0.001), while shorter or longer durations did not reach statistical significance. Sex-based subgroup analysis showed a significant within-group effect in females (Hedges’ g = 0.955, 95% CI [0.04–1.87], *p* = 0.040), though the difference between female-only and mixed-sex samples was not significant. These results suggest that intervention duration, and to some extent age and BMI, may influence the effectiveness of sleep interventions.

### Network meta-analysis

**Figure 4:**
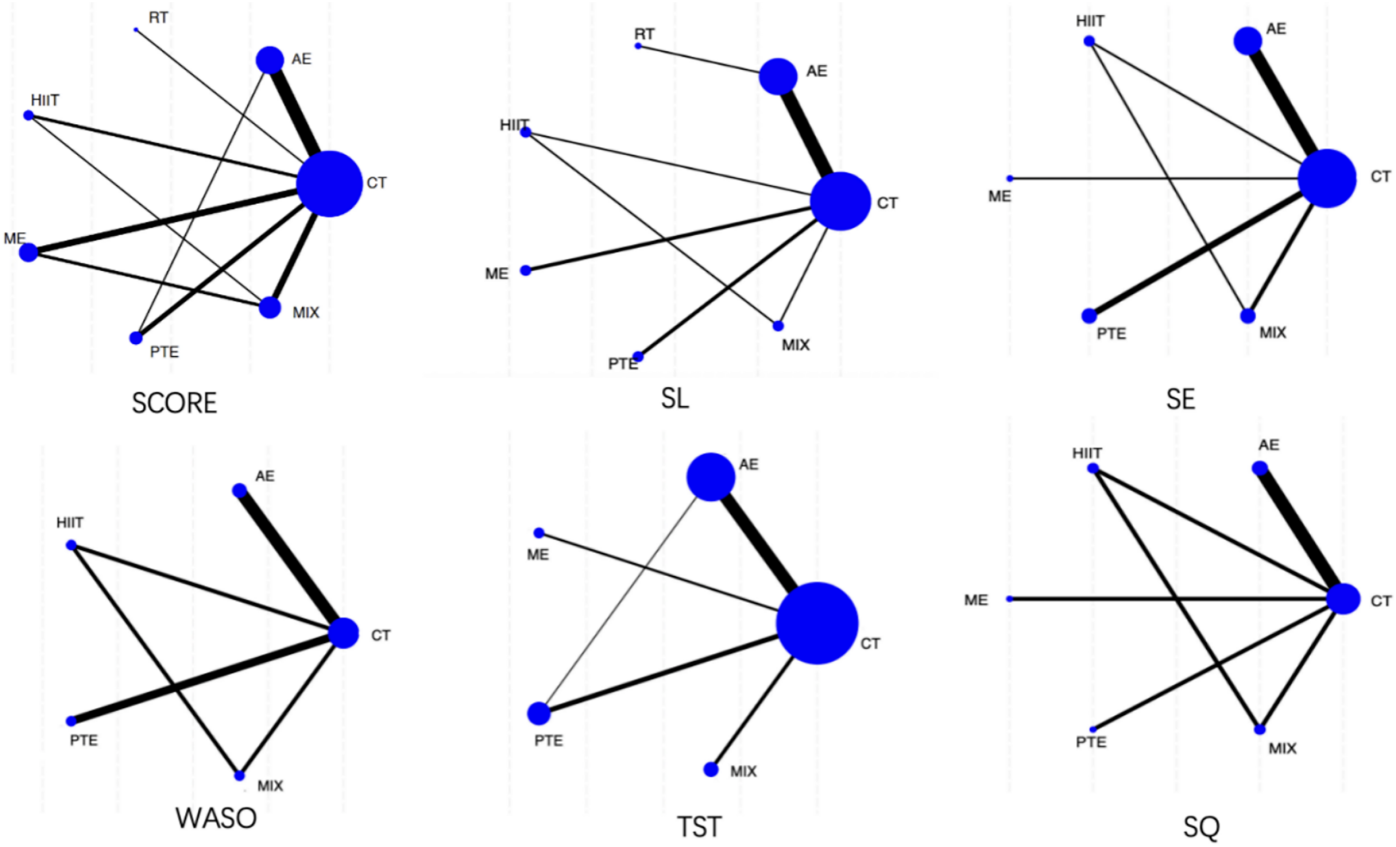
Network plot presenting the effects of different exercise modalities on sleep outcomes in sedentary populations. AE, Aerobic Exercise; RT, Resistance Training; HIIT, High-intensity Interval Training; ME, Mind-body Exercise; PTE, Prolonged Time of Exercise; MIX, Mixed Exercise; CG, Control Group; SCORE, sleep questionnaire score; SL, sleep latency; SE, sleep efficiency; TST, total sleep time; SQ, sleep quality; WASO, wake time after sleep onset. Node size is proportional to the total sample size for each intervention, and edge width is proportional to the number of studies directly comparing each pair of interventions.

Network diagrams (Fig. 4) depict the comparative relationships among exercise modalities for all sleep outcomes. The size of the nodes corresponds to the sample size of each exercise modality, while the thickness of the connecting lines reflects the number of studies comparing two specific modalities. AE was the most commonly compared exercise type, whereas RT was the least frequently studied. Nodes not directly connected represent indirect comparison evidence, while connected nodes indicate mixed comparison evidence. Node-splitting analyses supported local consistency across the network, as all comparisons showed no significant inconsistency between direct and indirect estimates. These results suggest that the transitivity assumption was adequately met for the outcomes assessed (Table S4). What’s more, the forest plots for network comparisons is shown in Fig. S2.

#### SCORE

A total of twenty-one studies reported total SCORE using subjective measurement tools, involving five different instruments: PSQI, ISI, FOSQ-10, Fantastic Life Questionnaire, and TAAQOL. Among these, sixteen studies utilized the PSQI, encompassing six types of interventions: AE, MIX, ME, PTE, HIIT, and RT. Two studies used the ISI, involving PTE and AE interventions, while one study employed the FOSQ-10 with the MIX intervention. The remaining two studies, both involving AE, used the Fantastic Life Questionnaire and TAAQOL, respectively. The inverted triangle plot results (Table 3) showed that PTE (Hedges’ g = 4.42, 95% CI [1.26–7.59]) demonstrated a significantly greater improvement in total SCORE on subjective measurement tools compared to CT. The SUCRA probability ranking (Fig. S3) further revealed the following order of effectiveness: PTE (SUCRA = 89.4%) > RT (SUCRA = 76.7%) > MIX (SUCRA = 46.6%) > ME (SUCRA = 46.0%) > HIIT (SUCRA = 44.3%) > AE (SUCRA = 38.6%) > CT (SUCRA = 8.5%).

**Table 3:** Net league table with network evidence.

	**CT**	1.60 (−0.47, 3.67)	4.42 (1.26, 7.59)	1.51 (−0.61, 3.63)	1.51 (−1.51, 4.52)	3.80 (−0.86, 8.46)	1.22 (−0.64, 3.08)	SCORE
**SL**	0.39 (−9.70, 10.48)	**MIX**	2.82 (−0.96, 6.60)	−0.09 (–2.53, 2.34)	−0.09 (−3.42, 3.23)	2.20 (−2.90, 7.30)	−0.38 (−3.16, 2.40)	
	−2.30 (−9.46, 4.86)	−2.69 (−15.06, 9.68)	**PTE**	−2.91 (−6.74, 0.91)	−2.92 (−7.29, 1.46)	−0.62 (−6.25, 5.01)	−3.20 (−6.86, 0.46)	
	5.40 (−1.95, 12.75)	5.01 (−7.47, 17.49)	7.70 (−2.56, 17.96)	**ME**	−0.00 (−3.58, 3.58)	2.29 (−2.83, 7.41)	−0.29 (−3.10, 2.53)	
	0.12 (−9.97, 10.21)	−0.27 (−10.35, 9.81)	2.42 (−9.95, 14.79)	−5.28 (−17.76, 7.20)	**HIIT**	2.29 (−3.26, 7.85)	−0.29 (−3.83, 3.26)	
	7.10 (−4.06, 18.25)	6.71 (−8.33, 21.75)	9.40 (−3.86, 22.65)	1.70 (−11.64, 15.03)	6.98 (−8.06, 22.02)	**RT**	−2.58 (−7.60, 2.44)	
	2.80 (−1.80, 7.40)	2.41 (−8.68, 13.50)	5.10 (−3.42, 13.61)	−2.60 (−11.24, 6.04)	2.68 (−8.41, 13.77)	−4.30 (−14.46, 5.86)	**AE**	
	**CT**	0.04 (−0.95, 1.03)	1.30 (−0.94, 3.54)	0.13 (−1.16, 1.42)	−0.30 (−1.33, 0.73)	0.58 (0.01, 1.15)		**SQ**
**SE**	−4.05 (−6.94, −1.17)	**MIX**	1.26 (−1.19, 3.71)	0.09 (−1.54, 1.72)	−0.34 (−1.26, 0.58)	0.54 (−0.61, 1.69)		
	0.23 (−0.75, 1.20)	4.28 (1.24, 7.33)	**PTE**	−1.17 (−3.76, 1.42)	−1.60 (−4.07, 0.87)	−0.72 (-3.03, 1.59)		
	−0.75 (−2.07, 0.57)	3.30 (0.13, 6.48)	−0.98 (−2.62, 0.66)	**ME**	−0.43 (−2.08, 1.22)	0.45 (−0.96, 1.86)		
	0.23 (−4.17, 4.63)	4.29 (0.19, 8.39)	0.01 (−4.50, 4.51)	0.98 (−3.61, 5.57)	**HIIT**	0.88 (−0.30, 2.06)		
	−1.29 (−2.45, −0.14)	2.76 (−0.38, 5.90)	−1.52 (−3.08, 0.03)	−0.54 (−2.30, 1.21)	−1.53 (−6.09, 3.04)	**AE**		
	**CT**	**MIX**	5.47 (−17.28, 28.22)	−3.63 (−21.46, 14.20)	−9.25 (−31.13, 12.63)			**WASO**
**TST**	−0.31 (−0.82, 0.19)	**MIX**	**PTE**	−9.10 (−31.74, 13.55)	−14.71 (−32.89, 3.46)			
	−0.19 (−0.87, 0.49)	0.12 (−0.72, 0.96)	**PTE**	**HIIT**	−5.62 (−27.39, 16.16)			
	−0.94 (−1.71, −0.17)	−0.63 (−1.55, 0.29)	−0.75 (−1.77, 0.27)	**ME**	**AE**			
	−0.77 (−1.23, −0.30)	−0.46 (−1.14, 0.23)	−0.58 (−1.40, 0.24)	0.17 (−0.73, 1.07)	**AE**			

**Note:**

AE, Aerobic Exercise; RT, Resistance Training; HIIT, High-Intensity Interval Training; ME, Mind-Body Exercise; PTE, Prolonged Time of Exercise; MIX, Mixed Exercise; CG, Control Group; SCORE, sleep questionnaire score; SL, sleep latency; SE, sleep efficiency; TST, total sleep time; SQ, sleep quality; WASO, wake time after sleep onset; All sleep outcomes were harmonized such that higher values indicate better sleep (*e.g*., PSQI, SL, and WASO).

#### SL

A total of eleven studies reported on SL, utilizing three measurement tools: PSQI, accelerometer, and PSG. Among these, seven studies used the PSQI, involving three types of interventions: AE, ME, and MIX. Three studies utilized accelerometers, encompassing AE, ME, and PTE interventions, while three studies employed PSG, all involving AE intervention. The inverted triangle plot (Table 3) results showed no statistically significant differences in the effects of various exercise modalities on SL improvement or in the comparative effectiveness between exercise types. The SUCRA probability ranking (Fig. S3) revealed the following order: PTE (SUCRA = 82.0%) > CT (SUCRA = 66.3%) > HIIT (SUCRA = 61.1%) > MIX (SUCRA = 57.5%) > AE (SUCRA = 40.3%) > ME (SUCRA = 24.2%) > RT (SUCRA = 18.7%).

#### SE

A total of twelve studies reported on SE, utilizing three measurement tools: PSQI, PSG, and accelerometer. Among these, five studies used the PSQI, involving three types of interventions: AE, ME, and PTE. Four studies employed PSG, all involving AE intervention, while four studies used accelerometers, encompassing PTE and MIX interventions. The inverted triangle plot (Table 3) results showed that, compared to the CT, MIX significantly improved SE (Hedges’ g = −4.05, 95% CI [−6.94 to −1.17]). Additionally, MIX demonstrated significantly better effects compared to PTE, ME, and HIIT. The SUCRA probability ranking (Fig. S3) revealed the following order: MIX (SUCRA = 98.3%) > AE (SUCRA = 69.7%) > ME (SUCRA = 53.2%) > HIIT (SUCRA = 32.9.7%) > PTE (SUCRA = 19.5%).

#### WASO

A total of five studies reported on WASO, utilizing two measurement tools: PSG and accelerometer. Among these, three studies used accelerometers, involving two types of interventions: AE, HIIT, MIX and PTE. The remaining three studies employed PSG, all involving AE intervention. The inverted triangle plot (Table 3) results showed no statistically significant differences in the effects of various exercise modalities on WASO improvement or in the comparative effectiveness between exercise types. The SUCRA probability ranking (Fig. S3) revealed the following order: PTE (SUCRA = 80.8%) > MIX (SUCRA = 58.5%) > CT (SUCRA = 50.0%) > HIIT (SUCRA = 41.7%) > AE (SUCRA = 19.0%)

#### TST

A total of eighteen studies reported on TST, utilizing five measurement tools: PSQI, accelerometer, PSG, and MARCA. Among these, ten studies used the PSQI, involving four types of interventions: AE, ME, MIX, and PTE. Eight studies employed accelerometers, encompassing three interventions: AE, PTE, and MIX. Four studies used PSG, all involving AE intervention, and one study utilized MARCA, also involving AE intervention. Additionally, AE (Hedges’ g = 0.77, 95% CI [0.30–1.23]) and ME (Hedges’ g = 0.94, 95% CI [0.17–1.71]) demonstrated significantly better effects compared to CT. The SUCRA probability ranking (Fig. S3) revealed the following order: ME (SUCRA = 86.6%) > AE (SUCRA = 80.1%) > MIX (SUCRA = 42.6%) > PTE (SUCRA = 29.8%) > CT (SUCRA = 10.8%).

#### SQ

A total of six studies reported on SQ, utilizing two measurement tools: PSQI and PSG. Among these, five studies used the PSQI, involving two types of interventions: AE and PTE. One study employed PSG, involving AE intervention. The inverted triangle plot (Table 3) results showed that the CT had a significantly negative effect relative to AE (Hedges’ g = 0.58, 95% CI: −1.15 to −0.01), indicating that CT was less effective than AE in improving SQ. The SUCRA probability ranking (Fig. S3) revealed the following order: PTE (SUCRA = 83.4%) > AE (SUCRA = 74.7%) > ME (SUCRA = 44.7%) > MIX (SUCRA = 42.3%) > CT (SUCRA = 34.3%) > HIIT (SUCRA = 20.6%).

## Discussion

This systematic review and NMA synthesized 31 RCTs involving 1,420 sedentary participants. First, a meta-analysis was conducted to assess the overall impact of physical activity on sleep improvement, revealing that exercise significantly improved SCORE. Next, subgroup analyses were performed to explore the effects of exercise on sleep outcomes across different age groups and BMI ranges. Subgroup analyses suggested greater benefits for middle-aged and overweight individuals, although between-group differences were not statistically significant. Finally, NMA was used to compare the differences and effect sizes of various exercise modalities on sleep improvement. We found that the following exercises had notable improvements on specific sleep outcomes: (1) PTE for SCORE, SE, SQ, WASO, and TST; (2) AE for SE, TST, and SQ; (3) MIX and ME for SE and TST.

While a recent NMA by Riedel et al. (2024) confirmed that exercise improves sleep in individuals with insomnia (SMD = 0.90 for subjective outcomes), and earlier reviews (Hasan et al., 2022) have highlighted the general benefits of physical activity, none provide a comparative effectiveness ranking across specific exercise modalities—particularly in sedentary adults without clinical insomnia, a population in which optimal intervention selection remains unclear. Our NMA provides preliminary evidence for a modality-specific hierarchy that may inform tailored exercise approaches in this population. First, we found that PTE significantly improved multiple sleep outcomes, especially subjective measures like the PSQI total score. This type of exercise focuses on reducing sedentary time and increasing overall physical activity duration without emphasizing specific exercise modalities. This improvement likely reflects enhanced mood and self-perceived well-being, as even minimal physical engagement can positively influence psychological states. In previous studies, researchers have demonstrated the positive effects of this activity pattern on emotional states, including valence, energetic arousal, and calmness (Giurgiu et al., 2020). Even engaging in 5-min breaks to interrupt sedentary behavior every 30 min or hourly can significantly reduce fatigue and emotional distress, with the latter primarily driven by an increase in vitality (Duran et al., 2023). While mechanisms remain incompletely understood, these breaks may support cardiovascular regulation, potentially contributing to better sleep. Notably, the reduction in systolic blood pressure (~3 to 5 mm Hg) following such activity is comparable to the hypotensive benefits observed after AE (Cardoso et al., 2010; Cornelissen & Smart, 2013). Chronic emotional stress can dysregulate both the hypothalamic-pituitary-adrenal, leading to sustained cortisol release and activation of the renin-angiotensin-aldosterone system—both of which contribute to elevated blood pressure and cardiovascular strain (Stanton, Price & Manning, 2023; Zeng et al., 2024). Moreover, stress-induced hyperactivity in emotion-processing brain regions (*e.g*., amygdala, prefrontal cortex) can directly modulate cardiovascular regulatory centers, further exacerbating physiological arousal (Grisk & Rettig, 2004; Jiang et al., 2021; Zeng et al., 2024). Together, these findings suggest a bidirectional relationship: light activity may improve sleep through mood enhancement and autonomic modulation, while better sleep, in turn, reinforces emotional and cardiovascular stability—a positive feedback loop supported by recent evidence (Gong et al., 2024; Jun et al., 2012).

Additionally, AE demonstrated significant benefits for SE, TST, and SQ in sedentary populations. In older adults, AE may counteract age-related disruptions in circadian regulation, a key factor in sleep deterioration. For example, 16 weeks of moderate-intensity AE improved sleep and well-being in older adults (Reid et al., 2010). Animal studies further suggest that AE enhances the amplitude of circadian rhythms, potentially restoring sleep-wake cycle stability (Leise et al., 2013). By stabilizing the body’s biological clock, exercise can enhance overall health. AE may also act through serotonergic pathways, as serotonin (5-HT) is a key modulator of both mood and sleep regulation, and its activity declines with age (Melancon, Lorrain & Dionne, 2014). These benefits extend beyond older adults: in younger sedentary populations, AE also consistently improves deep sleep and TST (Trinder et al., 1985). Beyond exercise type, exercise intensity is also a critical factor. Passos et al. (2010) found that a single session of moderate-intensity aerobic exercise significantly outperformed high-intensity exercise in improving SL, TST, and SE, while reducing anxiety levels in individuals with primary insomnia. This highlights moderate-intensity aerobic exercise as a practical, non-pharmacological intervention. Across the included trials, effective AE protocols typically involved moderate-to-vigorous intensity (60–80% HRmax), ~3 sessions/week, and ≥30 min/session (*e.g*., brisk walking, jogging, swimming)**—**reflecting common practice rather than a definitive dose-response prescription.

MIX and ME are also recommended exercise modalities. ME—including Tai Chi, Qigong, and yoga—have consistently demonstrated benefits for insomnia. Unlike AE, which primarily enhances cardiorespiratory fitness through elevated heart rate and sustained intensity, ME emphasizes slow, mindful movement integrated with breath regulation and meditation. This practice preferentially activates the parasympathetic nervous system, reduces sympathetic tone, and lowers heart rate and blood pressure, thereby counteracting the hyperarousal commonly observed in insomnia. Emerging evidence also suggests that ME may modulate neuroendocrine pathways, including downregulation of hypothalamic–pituitary–adrenal axis activity and reduced cortisol secretion (Gong et al., 2024; Li et al., 2020). Neuroimaging studies further support that Tai Chi can enhance functional connectivity between the default mode network and frontoparietal network, potentially optimizing cognitive control and reducing neural energy cost associated with fatigue and sleep disruption (Wu et al., 2022). MIX may offer complementary benefits through multimodal physiological engagement. While all exercise types likely influence neurotransmitter activity and energy balance—thereby increasing sleep drive—the optimal choice may depend on individual preference and feasibility. (Gong et al., 2024; Jun et al., 2012). While all exercise types likely enhance sleep drive *via* neurotransmitter and energy regulation, the optimal modality should be guided by individual preference and tolerability.

### Limitations and interpretation

This study has several limitations. First, the number of trials for certain exercise modalities was small, leading to imprecise effect estimates and unstable rankings. Second, we pooled subjective (*e.g*., PSQI) and objective (*e.g*., actigraphy) sleep outcomes to maximize data inclusion; while pragmatic, this may introduce measurement bias due to differences in assessment validity. Third, the limited number of studies constrained detailed subgroup analyses by key factors such as sex or ethnicity. Last, The clinical relevance of observed effects remains uncertain, as minimal clinically important difference (MCID) thresholds are not established for all instruments used; even for PSQI, MCID estimates vary across populations.

## Conclusion

Given the high global prevalence of sleep problems, structured exercise appears to be a promising non-pharmacological strategy for improving sleep in sedentary adults. This network meta-analysis found that different exercise modalities show differential benefits across specific sleep outcomes: (1) PTE was associated with improvements in SCORE, SE, and TST; (2) AE showed favorable effects on SE, TST, and SQ; and (3) MIX and ME were beneficial for SE and TST. These findings may inform the selection of exercise types tailored to individual needs and sleep profiles. However, optimal parameters such as session frequency, intensity, and timing require further investigation in dedicated trials.

## Supplemental Information

10.7717/peerj.21037/supp-1 Supplemental Information 1 Supplementary Materials.

10.7717/peerj.21037/supp-2 Supplemental Information 2 PRISMA checklist.

## Additional Information and Declarations

### Data Availability

The following information was supplied regarding data availability:

This is a systematic review/meta-analysis.

## References

[ref-1] Al-Jiffri OH, Abd El-Kader SM (2021). Aerobic versus resistance exercises on systemic inflammation and sleep parameters in obese subjects with chronic insomnia syndrome. African Health Sciences.

[ref-2] Baker BS, Weitzel KJ, Royse LA, Miller K, Guess TM, Ball SD, Duren DL (2021). Efficacy of an 8-week resistance training program in older adults: a randomized controlled trial. Journal of Aging & Physical Activity.

[ref-180] Barbosa WA, Leite CDFC, Reis CHO, Machado AF, Bullo V, Gobbo S, Bergamin M, Lima-Leopoldo AP, Vancini RL, Baker JS, Rica RL, Bocalini DS (2023). Effect of supervised and unsupervised exercise training in outdoor gym on the lifestyle of elderly people. International Journal of Environmental Research and Public Health.

[ref-3] Benca RM (2005). Diagnosis and treatment of chronic insomnia: a review. Psychiatric Services.

[ref-4] Boing L, Fretta TDB, Lynch BM, Dias M, Rosa LMD, Baptista F, Bergmann A, Fausto DY, Bocchi JB, Guimarães ACDA (2023). Mat Pilates and belly dance: effects on patient-reported outcomes among breast cancer survivors receiving hormone therapy and adherence to exercise. Complementary Therapies in Clinical Practice.

[ref-190] Brooker P, Gomersall S, King N, McMahon N, Leveritt M (2023). How do previously inactive individuals restructure their time to “fit in” morning or evening exercise: a randomized controlled trial. Journal of Behavioral Medicine.

[ref-5] Cardoso CG, Gomides RS, Carrenho Queiroz AC, Pinto LG, Lobo FdS, Tinucci T, Mion D, de Moraes Forjaz CL (2010). Acute and chronic effects of aerobic and resistance exercise on ambulatory blood pressure. Clinics.

[ref-6] Cornelissen VA, Smart NA (2013). Exercise training for blood pressure: a systematic review and meta-analysis. Journal of the American Heart Association.

[ref-7] Davidson P, Marcusson-Clavertz D (2023). The effect of sleep on intrusive memories in daily life: a systematic review and meta-analysis of trauma film experiments. Sleep.

[ref-49] de Jong J, Lemmink KAPM, Stevens M, de Greef MHG, Rispens P, King AC, Mulder T (2006). Six-month effects of the groningen active living model (GALM) on physical activity, health and fitness outcomes in sedentary and underactive older adults aged 55–65. Patient Education and Counseling.

[ref-8] Duran AT, Friel CP, Serafini MA, Ensari I, Cheung YK, Diaz KM (2023). Breaking up prolonged sitting to improve cardiometabolic risk: dose-response analysis of a randomized crossover trial. Medicine & Science in Sports & Exercise.

[ref-160] Durante BG, Ferreira-Silva R, Goya TT, Lima MF, Rodrigues ACT, Drager LF, Jordão CP, Rodrigues AG, Alves MJNN, Lorenzi-Filho G, Negrão CE, Ueno-Pardi LM (2022). Effects of exercise training on left ventricular diastolic function markers in patients with obstructive sleep apnea: a randomized study. International Journal of Cardiovascular Sciences.

[ref-55] El-Kader SMA, Al-Jiffri OH (2019). Aerobic exercise modulates cytokine profile and sleep quality in elderly. African Health Sciences.

[ref-9] Ford ES, Wheaton AG, Cunningham TJ, Giles WH, Chapman DP, Croft JB (2014). Trends in outpatient visits for insomnia, sleep apnea, and prescriptions for sleep medications among us adults: findings from the national ambulatory medical care survey 1999–2010. Sleep.

[ref-40] Frimpong E, Mograss M, Zvionow T, Dang-Vu TT (2021). The effects of evening high-intensity exercise on sleep in healthy adults: a systematic review and meta-analysis. Sleep Medicine Reviews.

[ref-10] Frye B, Scheinthal S, Kemarskaya T, Pruchno R (2007). Tai chi and low impact exercise: effects on the physical functioning and psychological well-being of older people. Journal of Applied Gerontology.

[ref-210] Gale JT, Haszard JJ, Wei DL, Taylor RW, Peddie MC (2024). Evening regular activity breaks extend subsequent free-living sleep time in healthy adults: a randomised crossover trial. BMJ Open Sport & Exercise Medicine.

[ref-11] García-Soidán JL, Giraldez VA, Zagalaz JC, Lara-Sánchez AJ (2014). Does Pilates exercise increase physical activity, quality of life, latency, and sleep quantity in middle-aged people?. Perceptual and Motor Skills.

[ref-12] Giurgiu M, Koch ED, Plotnikoff RC, Ebner-Priemer UW, Reichert M (2020). Breaking up sedentary behavior optimally to enhance mood. Medicine and Science in Sports and Exercise.

[ref-13] Gong M, Tang Q, Tan S, Hu X (2024). Research progress in the effect of exercise intervention on sleep disorders and the mechanisms involved. Sichuan Da Xue Xue Bao. Yi Xue Ban = Journal of Sichuan University. Medical Science Edition.

[ref-14] Grisk O, Rettig R (2004). Interactions between the sympathetic nervous system and the kidneys in arterial hypertension. Cardiovascular Research.

[ref-15] Guo J, Liu J, Zhu R, Liu G, Zheng M, Cao C (2024). The impact of different types of exercise on executive functions in overweight/obese individuals: a systematic review and network meta-analysis. Behavioral Sciences.

[ref-16] Hasan F, Tu Y-K, Lin C-M, Chuang L-P, Jeng C, Yuliana LT, Chen T-J, Chiu H-Y (2022). Comparative efficacy of exercise regimens on sleep quality in older adults: a systematic review and network meta-analysis. Sleep Medicine Reviews.

[ref-53] Hartescu I, Morgan K, Stevinson CD (2015). Increased physical activity improves sleep and mood outcomes in inactive people with insomnia: a randomized controlled trial. Journal of Sleep Research.

[ref-47] Hortobágyi T, Granacher U, Fernandez-del-Olmo M, Howatson G, Manca A, Deriu F, Taube W, Gruber M, Márquez G, Lundbye-Jensen J, Colomer-Poveda D (2021). Functional relevance of resistance training-induced neuroplasticity in health and disease. Neuroscience & Biobehavioral Reviews.

[ref-54] Hurdiel R, Watier T, Honn K, Pezé T, Zunquin G, Theunynck D (2017). Effects of a 12-week physical activities programme on sleep in female university students. Research in Sports Medicine.

[ref-17] Innes KE, Selfe TK (2012). The effects of a gentle yoga program on sleep, mood, and blood pressure in older women with Restless Legs Syndrome (RLS): a preliminary randomized controlled trial. Evidence-Based Complementary and Alternative Medicine.

[ref-42] Irwin MR, Olmstead R, Breen EC, Witarama T, Carrillo C, Sadeghi N, Arevalo JMG, Ma J, Nicassio P, Cole SW (2014). Tai chi, cellular inflammation, and transcriptome dynamics in breast cancer survivors with insomnia: a randomized controlled trial. Journal of the National Cancer Institute Monographs.

[ref-18] Jiang N, Xu J, Li X, Wang Y, Zhuang L, Qin S (2021). Negative parenting affects adolescent internalizing symptoms through alterations in amygdala-prefrontal circuitry: a longitudinal twin study. Biological Psychiatry.

[ref-19] Jun H, Mohammed Qasim Hussaini S, Rigby MJ, Jang M-H (2012). Functional role of adult hippocampal neurogenesis as a therapeutic strategy for mental disorders. Neural Plasticity.

[ref-110] Jurado-Fasoli L, De-la-O A, Molina-Hidalgo C, Migueles JH, Castillo MJ, Amaro-Gahete FJ (2020). Exercise training improves sleep quality: a randomized controlled trial. European Journal of Clinical Investigation.

[ref-20] King AC, Oman RF, Brassington GS, Bliwise DL, Haskell WL (1997). Moderate-intensity exercise and self-rated quality of sleep in older adults: a randomized controlled trial. JAMA: The Journal of the American Medical Association.

[ref-48] King AC, Baumann K, O’Sullivan P, Wilcox S, Castro C (2002). Effects of moderate-intensity exercise on physiological, behavioral, and emotional responses to family caregiving: a randomized controlled trial. The Journals of Gerontology: Series A, Biological Sciences and Medical Sciences.

[ref-100] King AC, Pruitt LA, Woo S, Castro CM, Ahn DK, Vitiello MV, Woodward SH, Bliwise DL (2008). Effects of moderate-intensity exercise on polysomnographic and subjective sleep quality in older adults with mild to moderate sleep complaints. The Journals of Gerontology: Series A, Biological Sciences and Medical Sciences.

[ref-50] Kline CE, Ewing GB, Burch JB, Blair SN, Durstine JL, Davis JM, Youngstedt SD (2012). Exercise training improves selected aspects of daytime functioning in adults with obstructive sleep apnea. Journal of Clinical Sleep Medicine.

[ref-21] Leise TL, Harrington ME, Molyneux PC, Song I, Queenan H, Zimmerman E, Lall GS, Biello SM (2013). Voluntary exercise can strengthen the circadian system in aged mice. AGE.

[ref-22] Li H, Chen J, Xu G, Duan Y, Huang D, Tang C, Liu J (2020). The Effect of Tai Chi for improving sleep quality: a systematic review and meta-analysis. Journal of Affective Disorders.

[ref-200] Lohman T, Bains G, Cole S, Gharibvand L, Berk L, Lohman E (2023). High-intensity interval training reduces transcriptomic age: a randomized controlled trial. Aging Cell.

[ref-23] Manifield J, Chaudhry Y, Singh S, Ward T, Whelan M, Orme M (2024). Changes in physical activity, sedentary behaviour and sleep following pulmonary rehabilitation: a systematic review and network meta-analysis. European Respiratory Review.

[ref-170] McDonough DJ, Helgeson MA, Liu W, Gao Z (2022). Effects of a remote, YouTube-delivered exercise intervention on young adults’ physical activity, sedentary behavior, and sleep during the COVID-19 pandemic: randomized controlled trial. Journal of Sport and Health Science.

[ref-24] Melancon MO, Lorrain D, Dionne IJ (2014). Exercise and sleep in aging: emphasis on serotonin. Pathologie Biologie.

[ref-25] Mello M, Esteves A, Lopes C, Tufik S (2007). The impact of complaints and sleep disorders in the quality of life. 21st Annual Meeting of the American-Professional-Sleep-Societies.

[ref-26] Mystakidou K, Parpa E, Tsilika E, Pathiaki M, Gennatas K, Smyrniotis V, Vassiliou I (2007). The relationship of subjective sleep quality, pain, and quality of life in advanced cancer patients. Sleep.

[ref-27] Nadarajah S, Akiba R, Maricar I, Vohra S, Jamal A, Yano Y, Srinivasan M, Kim G, Huang RJ, Palaniappan L, Kim K, Elfassy T, Yang E (2025). Association between sleep duration and cardiovascular disease among asian americans. Journal of the American Heart Association.

[ref-41] Oliveira J, Gentil P, Naves JP, Souza Filho LF, Silva L, Zamunér AR, de Lira CA, Rebelo A (2022). Effects of high intensity interval training versus sprint interval training on cardiac autonomic modulation in healthy women. International Journal of Environmental Research and Public Health.

[ref-51] Oudegeest-Sander MH, Eijsvogels THM, Verheggen RJHM, Poelkens F, Hopman MTE, Jones H, Thijssen DHJ (2012). Impact of physical fitness and daily energy expenditure on sleep efficiency in young and older humans. Gerontology.

[ref-28] Pan T, Zeng Y, Chai X, Wen Z, Tan X, Sun M (2024). Global prevalence of perinatal depression and its determinants among rural women: a systematic review and meta-analysis. Depression and Anxiety.

[ref-220] Passos GS, Poyares D, Santana MG, Garbuio SA, Tufik S, Mello MT (2010). Effect of acute physical exercise on patients with chronic primary insomnia. Journal of Clinical Sleep Medicine.

[ref-43] Quist JS, Blond MB, Gram AS, Steenholt CB, Janus C, Holst JJ, Rehfeld JF, Sjödin AM, Stallknecht B, Rosenkilde M (2019). Effects of active commuting and leisure-time exercise on appetite in individuals with overweight and obesity. Journal of Applied Physiology.

[ref-46] Rayward AT, Murawski B, Duncan MJ, Holliday EG, Vandelanotte C, Brown WJ, Plotnikoff RC (2020). Efficacy of an m-health physical activity and sleep intervention to improve sleep quality in middle-aged adults: the refresh study randomized controlled trial. Annals of Behavioral Medicine.

[ref-29] Reid KJ, Baron KG, Lu B, Naylor E, Wolfe L, Zee PC (2010). Aerobic exercise improves self-reported sleep and quality of life in older adults with insomnia. Sleep Medicine.

[ref-30] Riedel A, Benz F, Deibert P, Barsch F, Frase L, Johann AF, Riemann D, Feige B (2024). The effect of physical exercise interventions on insomnia: a systematic review and meta-analysis. Sleep Medicine Reviews.

[ref-140] Seol J, Lee J, Nagata K, Fujii Y, Joho K, Tateoka K, Inoue T, Liu J, Okura T (2021). Combined effect of daily physical activity and social relationships on sleep disorder among older adults: cross-sectional and longitudinal study based on data from the Kasama study. BMC Geriatrics.

[ref-31] Shrestha N, Kukkonen-Harjula KT, Verbeek JH, Ijaz S, Hermans V, Bhaumik S (2016). Workplace interventions for reducing sitting at work. The Cochrane Database of Systematic Reviews.

[ref-32] Slavish DC, Ruggero CJ, Luft B, Kotov R (2024). Sleep disturbances across 2 weeks predict future mental healthcare utilization. Sleep.

[ref-33] Stanton LM, Price AJ, Manning EE (2023). Hypothalamic corticotrophin releasing hormone neurons in stress-induced psychopathology: revaluation of synaptic contributions. Journal of Neuroendocrinology.

[ref-52] Sternfeld B, Guthrie KA, Ensrud KE, LaCroix AZ, Larson JC, Dunn AL, Anderson GL, Seguin RA, Carpenter JS, Newton KM, Reed SD, Freeman EW, Cohen LS, Joffe H, Roberts MB, Caan BJ, Hohensee C (2014). Efficacy of exercise for menopausal symptoms: a randomized controlled trial. Menopause.

[ref-150] Teychenne M, Abbott G, Stephens LD, Opie RS, Olander EK, Brennan L, van der Pligt P, Apostolopoulos M, Ball K (2021). Mums on the move: a pilot randomised controlled trial of a home-based physical activity intervention for mothers at risk of postnatal depression. Midwifery.

[ref-34] Tobaldini E, Fiorelli EM, Solbiati M, Costantino G, Nobili L, Montano N (2019). Short sleep duration and cardiometabolic risk: from pathophysiology to clinical evidence. Nature Reviews Cardiology.

[ref-35] Trinder J, Paxton S, Montgomery I, Fraser G (1985). Endurance as opposed to power training—their effect on sleep. Psychophysiology.

[ref-120] Wang F, Boros S (2020). Effects of a pedometer-based walking intervention on young adults’ sleep quality, stress and life satisfaction: randomized controlled trial. Journal of Bodywork and Movement Therapies.

[ref-45] Wang F, Boros S (2021). The effect of daily walking exercise on sleep quality in healthy young adults. Sport Sciences for Health.

[ref-36] Wu K, Li Y, Zou Y, Ren Y, Wang Y, Hu X, Wang Y, Chen C, Lu M, Xu L, Wu L, Li K (2022). Tai Chi increases functional connectivity and decreases chronic fatigue syndrome: a pilot intervention study with machine learning and fMRI analysis. PLOS ONE.

[ref-44] Yeung WF, Lai AYK, Ho FYY, Suen LKP, Chung KF, Ho JYS, Ho LM, Yu BYM, Chan LYT, Lam TH (2018). Effects of zero-time exercise on inactive adults with insomnia disorder: a pilot randomized controlled trial. Sleep Medicine.

[ref-37] You Y, Chen Y, Fang W, Li X, Wang R, Liu J, Ma X (2023). The association between sedentary behavior, exercise, and sleep disturbance: a mediation analysis of inflammatory biomarkers. Frontiers in Immunology.

[ref-38] Zeng Y, Xiong B, Gao H, Liu C, Chen C, Wu J, Qin S (2024). Cortisol awakening response prompts dynamic reconfiguration of brain networks in emotional and executive functioning. Proceedings of the National Academy of Sciences of the United States of America.

