# Supplementary material for: The impact of different types of exercise on sleep in sedentary populations: a systematic review and network meta-analysis
Source: PeerJ. 2026 Jun 9;14:e21037. doi: 10.7717/peerj.21037 (PMC13262546; doi:10.7717/peerj.21037)
Supplement: Supplemental Information 1 [file peerj-14-21037-s001.docx]

| **Content** | **Pages** |
| --- | --- |
| Table S1. PRISMA-NMA checklist. | 2-5 |
| Table S2. Search query. | 6 |
| Table S3. Quality assessment of included studies. | 7-10 |
| Figure S1. Funnel plots illustrating the distribution of main sleep outcome indicators. | 11 |
| Table S4. Node-splitting inconsistency results. | 12 |
| Figure S2. Forest plots for all pairwise and network comparisons. | 13 |
| Figure S3. The SUCRA probability ranking. | 14 |

| **Section and Topic** | **Item #** | **Checklist item** | **Location where item is reported** |
| --- | --- | --- | --- |
| **TITLE** | | |  |
| Title | 1 | Identify the report as a systematic review. | P1 |
| **ABSTRACT** | | |  |
| Abstract | 2 | See the PRISMA 2020 for Abstracts checklist. | P1 |
| **INTRODUCTION** | | |  |
| Rationale | 3 | Describe the rationale for the review in the context of existing knowledge. | P2 |
| Objectives | 4 | Provide an explicit statement of the objective(s) or question(s) the review addresses. | P2 |
| **METHODS** | | |  |
| Eligibility criteria | 5 | Specify the inclusion and exclusion criteria for the review and how studies were grouped for the syntheses. | P3 |
| Information sources | 6 | Specify all databases, registers, websites, organisations, reference lists and other sources searched or consulted to identify studies. Specify the date when each source was last searched or consulted. | P3 |
| Search strategy | 7 | Present the full search strategies for all databases, registers and websites, including any filters and limits used. | P2-3 |
| Selection process | 8 | Specify the methods used to decide whether a study met the inclusion criteria of the review, including how many reviewers screened each record and each report retrieved, whether they worked independently, and if applicable, details of automation tools used in the process. | FIGURE 1 |
| Data collection process | 9 | Specify the methods used to collect data from reports, including how many reviewers collected data from each report, whether they worked independently, any processes for obtaining or confirming data from study investigators, and if applicable, details of automation tools used in the process. | P4 |
| Data items | 10a | List and define all outcomes for which data were sought. Specify whether all results that were compatible with each outcome domain in each study were sought (e.g. for all measures, time points, analyses), and if not, the methods used to decide which results to collect. | P3 |
|  | 10b | List and define all other variables for which data were sought (e.g. participant and intervention characteristics, funding sources). Describe any assumptions made about any missing or unclear information. | P3 |
| Study risk of bias assessment | 11 | Specify the methods used to assess risk of bias in the included studies, including details of the tool(s) used, how many reviewers assessed each study and whether they worked independently, and if applicable, details of automation tools used in the process. | P3 |
| Effect measures | 12 | Specify for each outcome the effect measure(s) (e.g. risk ratio, mean difference) used in the synthesis or presentation of results. | P3 |
| Synthesis methods | 13a | Describe the processes used to decide which studies were eligible for each synthesis (e.g. tabulating the study intervention characteristics and comparing against the planned groups for each synthesis (item #5)). | P4 |
|  | 13b | Describe any methods required to prepare the data for presentation or synthesis, such as handling of missing summary statistics, or data conversions. | P4 |
|  | 13c | Describe any methods used to tabulate or visually display results of individual studies and syntheses. | P4 |
|  | 13d | Describe any methods used to synthesize results and provide a rationale for the choice(s). If meta-analysis was performed, describe the model(s), method(s) to identify the presence and extent of statistical heterogeneity, and software package(s) used. | P4 |
|  | 13e | Describe any methods used to explore possible causes of heterogeneity among study results (e.g. subgroup analysis, meta-regression). | P4 |
|  | 13f | Describe any sensitivity analyses conducted to assess robustness of the synthesized results. | P4 |
| Reporting bias assessment | 14 | Describe any methods used to assess risk of bias due to missing results in a synthesis (arising from reporting biases). | - |
| Certainty assessment | 15 | Describe any methods used to assess certainty (or confidence) in the body of evidence for an outcome. | P4 |
| **RESULTS** | | |  |
| Study selection | 16a | Describe the results of the search and selection process, from the number of records identified in the search to the number of studies included in the review, ideally using a flow diagram. | P4 |
|  | 16b | Cite studies that might appear to meet the inclusion criteria, but which were excluded, and explain why they were excluded. | P4 |
| Study characteristics | 17 | Cite each included study and present its characteristics. | P4;TABLE 1 |
| Risk of bias in studies | 18 | Present assessments of risk of bias for each included study. | P4;TABLE S1 |
| Results of individual studies | 19 | For all outcomes, present, for each study: (a) summary statistics for each group (where appropriate) and (b) an effect estimate and its precision (e.g. confidence/credible interval), ideally using structured tables or plots. | P4;FIGURE 2;FIGURE3 |
| Results of syntheses | 20a | For each synthesis, briefly summarise the characteristics and risk of bias among contributing studies. | P4 |
|  | 20b | Present results of all statistical syntheses conducted. If meta-analysis was done, present for each the summary estimate and its precision (e.g. confidence/credible interval) and measures of statistical heterogeneity. If comparing groups, describe the direction of the effect. | P4 |
|  | 20c | Present results of all investigations of possible causes of heterogeneity among study results. | P4-5 |
|  | 20d | Present results of all sensitivity analyses conducted to assess the robustness of the synthesized results. | - |
| Reporting biases | 21 | Present assessments of risk of bias due to missing results (arising from reporting biases) for each synthesis assessed. | TABLE S1 |
| Certainty of evidence | 22 | Present assessments of certainty (or confidence) in the body of evidence for each outcome assessed. | TABLE S2 |
| **DISCUSSION** | | |  |
| Discussion | 23a | Provide a general interpretation of the results in the context of other evidence. | P7-8 |
|  | 23b | Discuss any limitations of the evidence included in the review. | P9 |
|  | 23c | Discuss any limitations of the review processes used. | P9 |
|  | 23d | Discuss implications of the results for practice, policy, and future research. | P7-8 |
| **OTHER INFORMATION** | | |  |
| Registration and protocol | 24a | Provide registration information for the review, including register name and registration number, or state that the review was not registered. | P2 |
|  | 24b | Indicate where the review protocol can be accessed, or state that a protocol was not prepared. | P2 |
|  | 24c | Describe and explain any amendments to information provided at registration or in the protocol. | - |
| Support | 25 | Describe sources of financial or non-financial support for the review, and the role of the funders or sponsors in the review. | TITLE PAGE |
| Competing interests | 26 | Declare any competing interests of review authors. | TITLE PAGE |
| Availability of data, code and other materials | 27 | Report which of the following are publicly available and where they can be found: template data collection forms; data extracted from included studies; data used for all analyses; analytic code; any other materials used in the review. | - |

**Table S1. PRISMA-NMA checklist**

|  | Search words | Search query (e.g. Pubmed) |
| --- | --- | --- |
| 1 | sedentary, inactive, desk-bound | Search: ((((sedentary[Title/Abstract]) OR (inactive[Title/Abstract])) OR (desk- bound[Title/Abstract])) AND ((((((((sleep[Title/Abstract]) OR (sleep quality[Title/Abstract])) OR (Sleep Onset Latency[Title/Abstract])) OR (Total sleep time[Title/Abstract])) OR (Fragmentation index[Title/Abstract])) OR (insomnia[Title/Abstract])) OR (Wake After Sleep Onset[Title/Abstract])) OR (Sleep Efficiency[Title/Abstract]))) AND ((((((((((((((exercise[Title/Abstract]) OR (physical activity[Title/Abstract])) OR (training[Title/Abstract])) OR (swimming[Title/Abstract])) OR (yoga[Title/Abstract])) OR (walking[Title/Abstract])) OR (aerobic[Title/Abstract])) OR (climbing[Title/Abstract])) OR (running[Title/Abstract])) OR (cycling[Title/Abstract])) OR (bicycling[Title/Abstract])) OR (resistance training[Title/Abstract])) OR (endurance training[Title/Abstract])) OR (Pilates[Title/Abstract])) Filters: Randomized Controlled Trial |
| 2 | exercise, physical activity, training, swimming, yoga, walking, aerobic, climbing, running, cycling, bicycling, resistance training, endurance training, pilates |  |
| 3 | sleep, sleep quality, sleep Onset Latency, total sleep time, fragmentation index, wake after sleep onset, sleep efficiency |  |
| 4 | Randomized controlled trail |  |

**Table S2. Search query**

| Jadad | | | | | | |
| --- | --- | --- | --- | --- | --- | --- |
|  | Study;country;region | Randomization | Concealment of allocation | Double blinding | Withdrawals and dropouts | Total |
| 1 | King et al.,1997;USA[1] | 2 | 0 | 0 | 1 | 3 |
| 2 | King et al.,2002;USA[2] | 2 | 0 | 0 | 1 | 3 |
| 3 | de Jong et al.,2006;USA[3] | 1 | 0 | 0 | 1 | 2 |
| 4 | Frye et al.,2007;USA[4] | 1 | 0 | 0 | 1 | 2 |
| 5 | King et al.,2008;USA[5] | 1 | 0 | 1 | 1 | 3 |
| 6 | Reid et a.,2010;USA[6] | 2 | 0 | 0 | 1 | 3 |
| 7 | Innes et al.,2012;USA[7] | 2 | 2 | 1 | 1 | 6 |
| 8 | Kline et al.,2012;USA[8] | 2 | 2 | 1 | 1 | 6 |
| 9 | Oudegeest-Sander et al.,2012;UK[9] | 1 | 0 | 0 | 1 | 2 |
| 10 | GARCÍA-SOIDÁN et al.,2014;UK[10] | 1 | 0 | 0 | 1 | 2 |
| 11 | Sternfeld et al.,2014;USA[11] | 2 | 1 | 1 | 1 | 5 |
| 12 | Hartescu et al.,2015;Spain[12] | 2 | 1 | 1 | 1 | 5 |
| 13 | Hurdiel et al.,2017;USA[13] | 1 | 0 | 0 | 1 | 2 |
| 14 | Yeung et al.,2018;China[14] | 2 | 2 | 1 | 1 | 6 |
| 15 | El-Kader and Al-Jiffri, 2019;Saudi Arabia[15] | 1 | 0 | 0 | 1 | 2 |
| 16 | Quist et al.,2019;Denmark[16] | 2 | 1 | 0 | 1 | 4 |
| 17 | Jurado-Fasoli et al.,2020;Spain[17] | 2 | 1 | 1 | 1 | 5 |
| 18 | Rayward et al.,2020;Australia[18] | 2 | 2 | 0 | 1 | 5 |
| 19 | Wang et al.,2020;Hungary[19] | 2 | 2 | 0 | 1 | 5 |
| 20 | Al-Jiffri and El-Kader , 2021;Saudi Arabia[20] | 1 | 0 | 0 | 1 | 2 |
| 21 | Baker et al.,2021;USA[21] | 2 | 1 | 1 | 1 | 5 |
| 22 | Hortobágyi et al.,2021;USA[22] | 2 | 1 | 1 | 1 | 6 |
| 23 | Seol et al.,2021;Japan[23] | 2 | 0 | 0 | 1 | 3 |
| 24 | Teychenne et al.,2021;UK[24] | 2 | 2 | 0 | 1 | 5 |
| 25 | Durante et al.,2022;Brazil[25] | 1 | 0 | 1 | 1 | 3 |
| 26 | McDonough et al.,2022;USA[26] | 1 | 0 | 1 | 1 | 3 |
| 27 | Barbosa et al.,2023;Brazil[27] | 2 | 0 | 0 | 1 | 3 |
| 28 | Boing et al.,2023;Brazil[28] | 2 | 0 | 0 | 1 | 3 |
| 29 | Brooker et al.,2023;Australia[29] | 2 | 0 | 1 | 1 | 4 |
| 30 | Lohman et al.,2023;USA[30] | 2 | 0 | 1 | 1 | 4 |
| 31 | Gale et al.,2024;Australia[31] | 2 | 2 | 0 | 1 | 5 |

**Table S3. Quality assessment of included studies.**

Randomization:

0:not randomized or inappropriate method of randomization;

1: the study was described as randomized;

2:the method of randomization was described and it was appropriate.

Concealment of allocation:

0: Not describe the method of allocation concealment;

1: The study was described as using allocation concealment method;

2: The method of allocation concealment was described appropriately.

Double blinding:

0: No blind or inappropriate method of blinding;

1: The study was described as double blind;

2: The method of double blinding was described and it was appropriate.

Withdrawals and dropouts:

0: Not describe the follow-up;

1: A description of withdrawals and dropouts.

**Included researches bibliography**

1. King, A.C.; Oman, R.F.; Brassington, G.S.; Bliwise, D.L.; Haskell, W.L. Moderate-Intensity Exercise and Self-Rated Quality of Sleep in Older Adults: A Randomized Controlled Trial. *JAMA* **1997**, *277*, 32–37, doi:10.1001/jama.277.1.32.

2. King, A.C.; Baumann, K.; O’Sullivan, P.; Wilcox, S.; Castro, C. Effects of Moderate-Intensity Exercise on Physiological, Behavioral, and Emotional Responses to Family Caregiving: A Randomized Controlled Trial. *The Journals of Gerontology Series A: Biological Sciences and Medical Sciences* **2002**, *57*, M26–M36, doi:10.1093/gerona/57.1.M26.

3. de Jong, J.; Lemmink, K.A.P.M.; Stevens, M.; de Greef, M.H.G.; Rispens, P.; King, A.C.; Mulder, T. Six-Month Effects of the Groningen Active Living Model (GALM) on Physical Activity, Health and Fitness Outcomes in Sedentary and Underactive Older Adults Aged 55-65. *Patient Educ Couns* **2006**, *62*, 132–141, doi:10.1016/j.pec.2005.06.017.

4. Frye, B.; Scheinthal, S.; Kemarskaya, T.; Pruchno, R. Tai Chi and Low Impact Exercise: Effects on the Physical Functioning and Psychological Well-Being of Older People. *Journal of Applied Gerontology* **2007**, *26*, 433–453, doi:10.1177/0733464807306915.

5. King, A.C.; Pruitt, L.A.; Woo, S.; Castro, C.M.; Ahn, D.K.; Vitiello, M.V.; Woodward, S.H.; Bliwise, D.L. Effects of Moderate-Intensity Exercise on Polysomnographic and Subjective Sleep Quality in Older Adults with Mild to Moderate Sleep Complaints. *Journals of Gerontology - Series A Biological Sciences and Medical Sciences* **2008**, *63*, 997–1004, doi:10.1093/gerona/63.9.997.

6. Reid, K.J.; Baron, K.G.; Lu, B.; Naylor, E.; Wolfe, L.; Zee, P.C. Aerobic Exercise Improves Self-Reported Sleep and Quality of Life in Older Adults with Insomnia. *Sleep Medicine* **2010**, *11*, 934–940, doi:10.1016/j.sleep.2010.04.014.

7. Innes, K.; Selfe, T. The Effects of a Gentle Yoga Program on Sleep, Mood, and Blood Pressure in Older Women with Restless Legs Syndrome (RLS): A Preliminary Randomized Controlled Trial. *EVIDENCE-BASED COMPLEMENTARY AND ALTERNATIVE MEDICINE* **2012**, *2012*, doi:10.1155/2012/294058.

8. Kline, C.E.; Ewing, G.B.; Burch, J.B.; Blair, S.N.; Durstine, J.L.; Davis, J.M.; Youngstedt, S.D. Exercise Training Improves Selected Aspects of Daytime Functioning in Adults with Obstructive Sleep Apnea. *Journal of Clinical Sleep Medicine* **2012**, *8*, 357–365, doi:10.5664/jcsm.2022.

9. Oudegeest-Sander, M.H.; Eijsvogels, T.H.M.; Verheggen, R.J.H.M.; Poelkens, F.; Hopman, M.T.E.; Jones, H.; Thijssen, D.H.J. Impact of Physical Fitness and Daily Energy Expenditure on Sleep Efficiency in Young and Older Humans. *Gerontology* **2012**, *59*, 8–16, doi:10.1159/000342213.

10. GARCÍA-SOIDÁN, J.L.; ARUFE GIRALDEZ, V.; CACHÓN ZAGALAZ, J.; LARA-SÁNCHEZ, A.J. DOES PILATES EXERCISE INCREASE PHYSICAL ACTIVITY, QUALITY OF LIFE, LATENCY, AND SLEEP QUANTITY IN MIDDLE-AGED PEOPLE? *Perceptual & Motor Skills* **2014**, *119*, 838–850, doi:10.2466/29.25.PMS.119c30z9.

11. Sternfeld, B.; Guthrie, K.A.; Ensrud, K.E.; Lacroix, A.Z.; Larson, J.C.; Dunn, A.L.; Anderson, G.L.; Seguin, R.A.; Carpenter, J.S.; Newton, K.M.; et al. Efficacy of Exercise for Menopausal Symptoms: A Randomized Controlled Trial. *Menopause* **2014**, *21*, 330–338, doi:10.1097/GME.0b013e31829e4089.

12. Hartescu, I.; Morgan, K.; Stevinson, C.D. Increased Physical Activity Improves Sleep and Mood Outcomes in Inactive People with Insomnia: A Randomized Controlled Trial. *Journal of sleep research* **2015**, *24*, 526–534, doi:10.1111/jsr.12297.

13. Hurdiel, R.; Watier, T.; Honn, K.; Pezé, T.; Zunquin, G.; Theunynck, D. Effects of a 12-Week Physical Activities Programme on Sleep in Female University Students. *Research in Sports Medicine* **2017**, *25*, 191–196, doi:10.1080/15438627.2017.1282354.

14. Yeung, W.-F.; Lai, A.Y.-K.; Ho, F.Y.-Y.; Suen, L.K.-P.; Chung, K.-F.; Ho, J.Y.-S.; Ho, L.-M.; Yu, B.Y.-M.; Chan, L.Y.-T.; Lam, T.-H. Effects of Zero-Time Exercise on Inactive Adults with Insomnia Disorder: A Pilot Randomized Controlled Trial. *Sleep Medicine* **2018**, *52*, 118–127, doi:10.1016/j.sleep.2018.07.025.

15. El-Kader, S.M.A.; Al-Jiffri, O.H. Aerobic Exercise Modulates Cytokine Profile and Sleep Quality in Elderly. *African Health Sciences* **2019**, *19*, 2198–2207, doi:10.4314/ahs.v19i2.45.

16. Quist, J.S.; Rosenkilde, M.; Gram, A.S.; Blond, M.B.; Holm-Petersen, D.; Hjorth, M.F.; Stallknecht, B.; Sjödin, A. Effects of Exercise Domain and Intensity on Sleep in Women and Men with Overweight and Obesity. *Journal of Obesity* **2019**, *2019*, doi:10.1155/2019/2189034.

17. Jurado-Fasoli, L.; De-la-O, A.; Molina-Hidalgo, C.; Migueles, J.H.; Castillo, M.J.; Amaro-Gahete, F.J. Exercise Training Improves Sleep Quality: A Randomized Controlled Trial. *European Journal of Clinical Investigation* **2020**, *50*, doi:10.1111/eci.13202.

18. Rayward, A.T.; Murawski, B.; Duncan, M.J.; Holliday, E.G.; Vandelanotte, C.; Brown, W.J.; Plotnikoff, R.C. Efficacy of an M-Health Physical Activity and Sleep Intervention to Improve Sleep Quality in Middle-Aged Adults: The Refresh Study Randomized Controlled Trial. *Annals of Behavioral Medicine* **2020**, *54*, 470–483, doi:10.1093/abm/kaz064.

19. Wang, F.; Boros, S. Effects of a Pedometer-Based Walking Intervention on Young Adults’ Sleep Quality, Stress and Life Satisfaction: Randomized Controlled Trial. *Journal of Bodywork and Movement Therapies* **2020**, *24*, 286–292, doi:10.1016/j.jbmt.2020.07.011.

20. Al-Jiffri, O.H.; Abd El-Kader, S.M. Aerobic versus Resistance Exercises on Systemic Inflammation and Sleep Parameters in Obese Subjects with Chronic Insomnia Syndrome. *African Health Sciences* **2021**, *21*, 1214–1222, doi:10.4314/ahs.v21i3.30.

21. Baker, B.S.; Weitzel, K.J.; Royse, L.A.; Miller, K.; Guess, T.M.; Ball, S.D.; Duren, D.L. Efficacy of an 8-Week Resistance Training Program in Older Adults: A Randomized Controlled Trial. *Journal of Aging & Physical Activity* **2021**, *29*, 121–129, doi:10.1123/japa.2020-0078.

22. Hortobágyi, T.; Deák, D.; Farkas, D.; Blényesi, E.; Török, K.; Granacher, U.; Tollár, J. Effects of Exercise Dose and Detraining Duration on Mobility at Late Midlife: A Randomized Clinical Trial. *Gerontology* **2021**, *67*, 403–414, doi:10.1159/000513505.

23. Seol, J.; Lee, J.; Nagata, K.; Fujii, Y.; Joho, K.; Tateoka, K.; Inoue, T.; Liu, J.; Okura, T. Combined Effect of Daily Physical Activity and Social Relationships on Sleep Disorder among Older Adults: Cross-Sectional and Longitudinal Study Based on Data from the Kasama Study. *BMC GERIATRICS* **2021**, *21*, doi:10.1186/s12877-021-02589-w.

24. Teychenne, M.; Abbott, G.; Stephens, L.D.; Opie, R.S.; Olander, E.K.; Brennan, L.; van der Pligt, P.; Apostolopoulos, M.; Ball, K. Mums on the Move: A Pilot Randomised Controlled Trial of a Home-Based Physical Activity Intervention for Mothers at Risk of Postnatal Depression. *Midwifery* **2021**, *93*, doi:10.1016/j.midw.2020.102898.

25. Durante, B.G.; Ferreira-Silva, R.; Goya, T.T.; Lima, M.F.; Rodrigues, A.C.T.; Drager, L.F.; Jordão, C.P.; Rodrigues, A.G.; Alves, M.J.N.N.; Lorenzi-Filho, G.; et al. Effects of Exercise Training on Left Ventricular Diastolic Function Markers in Patients with Obstructive Sleep Apnea: A Randomized Study. *International Journal of Cardiovascular Sciences* **2022**, *35*, 646–656, doi:10.36660/ijcs.20210146.

26. McDonough, D.J.; Helgeson, M.A.; Liu, W.; Gao, Z. Effects of a Remote, YouTube-Delivered Exercise Intervention on Young Adults’ Physical Activity, Sedentary Behavior, and Sleep during the COVID-19 Pandemic: Randomized Controlled Trial. *Journal of Sport and Health Science* **2022**, *11*, 145–156, doi:10.1016/j.jshs.2021.07.009.

27. Barbosa, W.A.; Leite, C.D.F.C.; Reis, C.H.O.; Machado, A.F.; Bullo, V.; Gobbo, S.; Bergamin, M.; Lima-Leopoldo, A.P.; Vancini, R.L.; Baker, J.S.; et al. Effect of Supervised and Unsupervised Exercise Training in Outdoor Gym on the Lifestyle of Elderly People. *Int J Environ Res Public Health* **2023**, *20*, doi:10.3390/ijerph20217022.

28. Boing, L.; Fretta, T.D.B.; Lynch, B.M.; Dias, M.; Rosa, L.M.D.; Baptista, F.; Bergmann, A.; Fausto, D.Y.; Bocchi, J.B.; Guimarães, A.C.D.A. Mat Pilates and Belly Dance: Effects on Patient-Reported Outcomes among Breast Cancer Survivors Receiving Hormone Therapy and Adherence to Exercise. *Complementary Therapies in Clinical Practice* **2023**, *50*, doi:10.1016/j.ctcp.2022.101683.

29. Brooker, P.; Gomersall, S.; King, N.; McMahon, N.; Leveritt, M. How Do Previously Inactive Individuals Restructure Their Time to “fit in” Morning or Evening Exercise: A Randomized Controlled Trial. *JOURNAL OF BEHAVIORAL MEDICINE* **2023**, *46*, 429–439, doi:10.1007/s10865-022-00370-x.

30. Lohman, T.; Bains, G.; Cole, S.; Gharibvand, L.; Berk, L.; Lohman, E. High-Intensity Interval Training Reduces Transcriptomic Age: A Randomized Controlled Trial. *Aging Cell* **2023**, *22*, doi:10.1111/acel.13841.

31. Gale, J.T.; Haszard, J.J.; Wei, D.L.; Taylor, R.W.; Peddie, M.C. Evening Regular Activity Breaks Extend Subsequent Free-Living Sleep Time in Healthy Adults: A Randomised Crossover Trial. *BMJ Open Sport Exerc Med* **2024**, *10*, e001774, doi:10.1136/bmjsem-2023-001774.


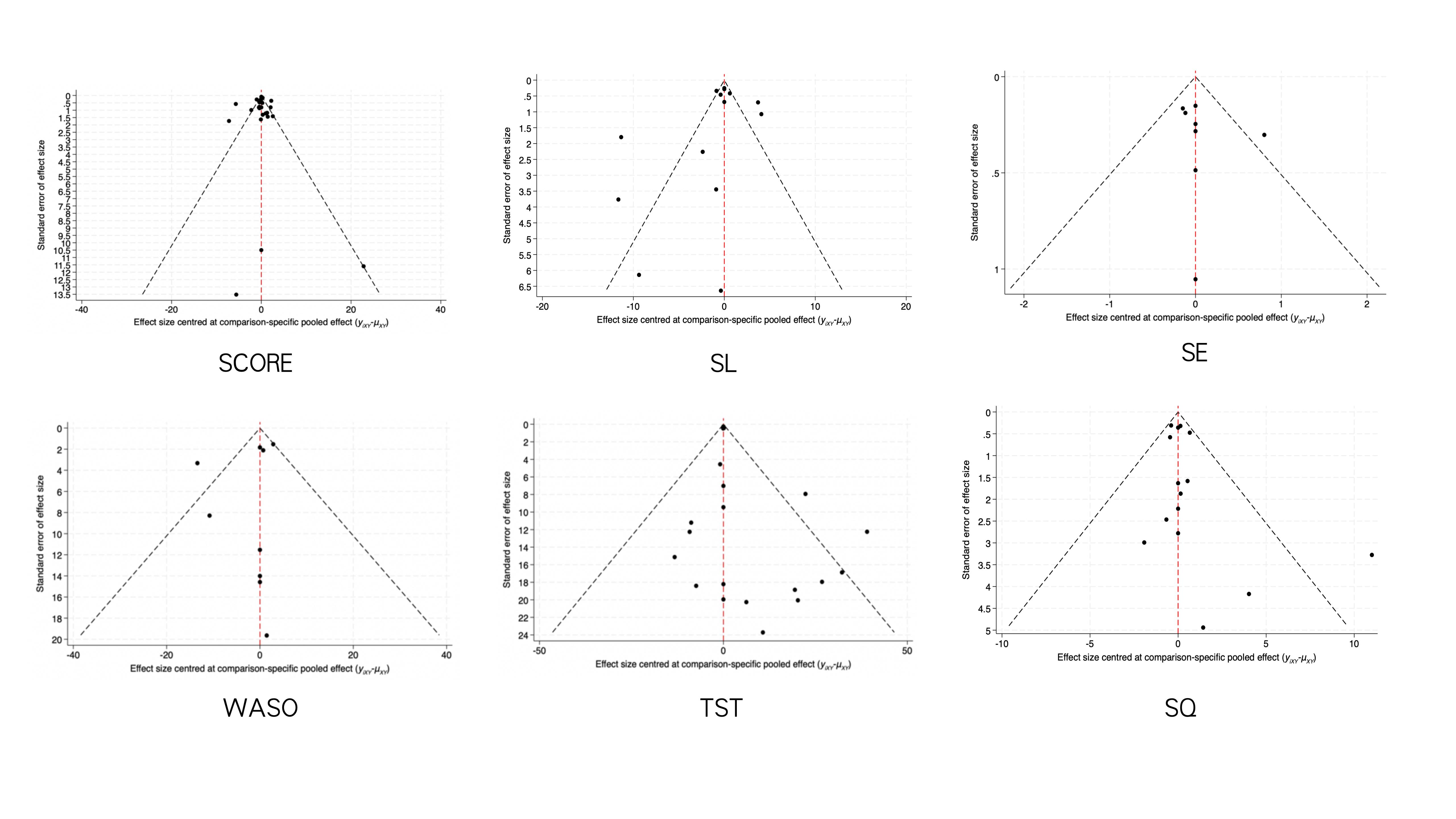


**Figure S1. Funnel plots illustrating the distribution of main sleep outcome indicators.** SCORE: Sleep questionnaire score; SL: Sleep latency; SE: Sleep efficiency; WASO: Wake after sleep onset; TST: Total sleep time; SQ: Sleep quality.

| Comparison | Direct (95% CI) | Indirect (95% CI) | Inconsistency Factor (95% CI) | p-value |
| --- | --- | --- | --- | --- |
| SCORE | | | | |
| AE vs RT | 1.09 (–0.75, 2.93) | 1.07 (-2.73, 4.87) | 0.02 (–4.21, 4.25) | 0.41 |
| AE vs ME | 1.35 (–1.98, 4.67) | 2.86 (–6.88, 12.60) | –1.52 (–11.83, 8.80) | 0.774 |
| AE vs PTE | 1.88 (–0.54, 4.31) | –0.09 (–5.14, 4.97) | 1.97 (–3.65, 7.59) | 0.492 |
| AE vs MIX | 3.1(-1.78, 7.98) | 0.87(–8.74, 10.48) | 2.23(-8.55, 13.01) | 0.22 |
| AE vs CT | 1.29 (–1.12, 3.69) | 2.83 (–1.93, 7.60) | –1.55 (–6.88, 3.79) | 0.571 |
| RT vs MIX | 0.62(–5.33, 4.09) | 3.61 (-0.43, 7.65) | -2.99 (–9.66, 7.84) | 0.83 |
| ME vs CT | –0.19 (–5.05, 4.68) | 0.39 (–4.61, 5.39) | –0.58 (–7.56, 6.40) | 0.871 |
| PTE vs CT | 0.54 (–2.83, 3.90) | –0.51 (–4.36, 3.34) | 1.05 (–4.07, 6.16) | 0.688 |
| SE | | | | |
| CT vs HIIT | –1.46 (–7.02, 4.10) | 3.46 (–7.70, 14.62) | –4.92 (–18.57, 8.73) | 0.480 |
| HIIT vs MIX | 3.63 (–0.86, 8.12) | 8.55 (–3.97, 21.07) | –4.92 (–18.57, 8.73) | 0.480 |
| TST | | | | |
| CT vs AE | 0.77 (0.30, 1.23) | 6.13 (–29.2, 41.5) | –5.37 (–40.6, 29.9) | 0.766 |
| CT vs PTE | 0.19 (–0.49, 0.87) | 6.32 (–25.9, 38.5) | –6.13 (–38.4, 26.1) | 0.71 |
| AE vs PTE | –0.04 (–18.4, 18.3) | –0.58 (–1.40, 0.24) | 0.53 (–17.8, 18.9) | 0.955 |

**Table S4. Node-splitting inconsistency results**


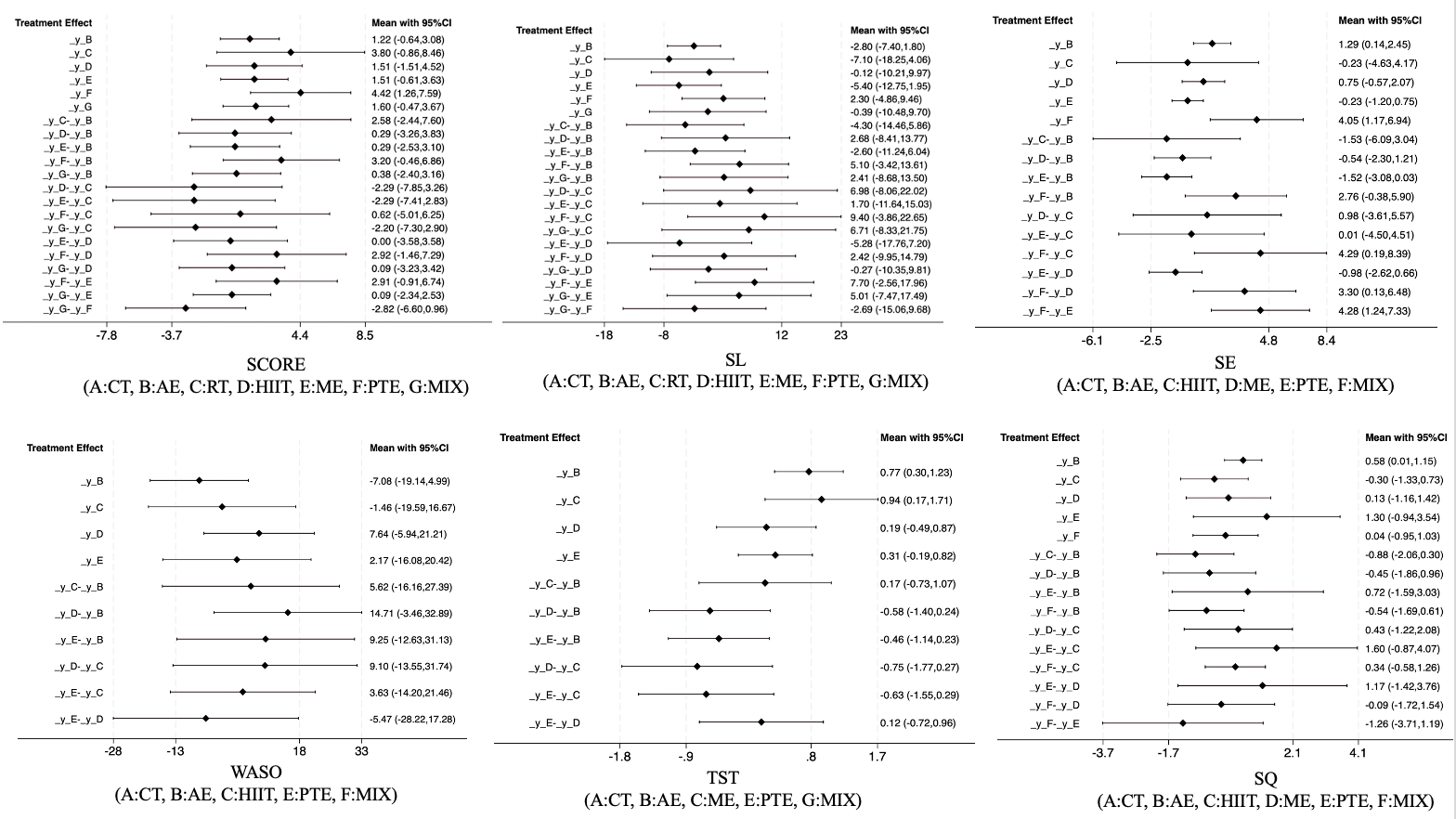


**Figure S2. Forest plots for network comparison**


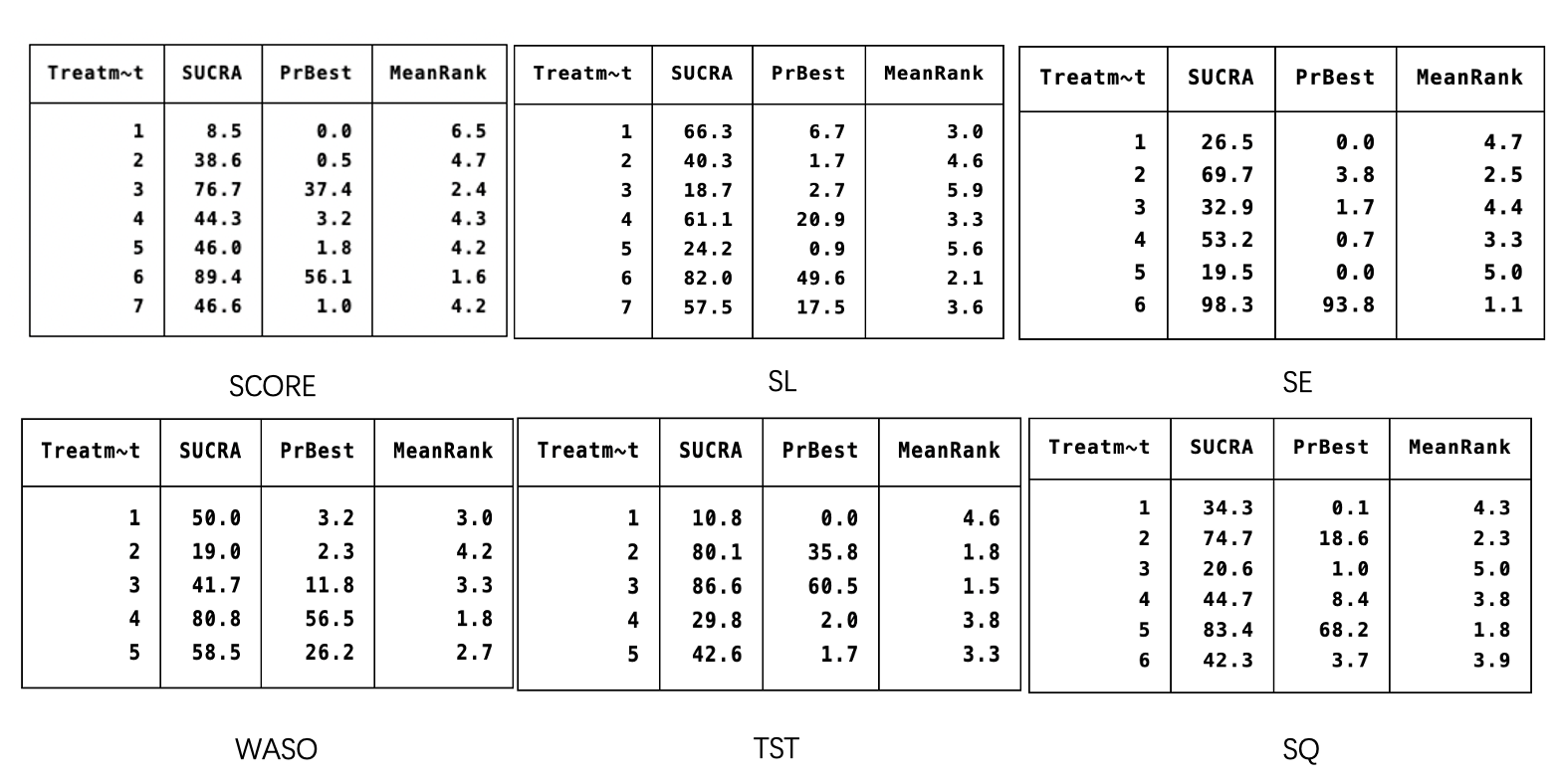


**Figure S3. Ranking results of main sleep outcome indicators.** Treatm~t: Intervention number; SUCRA: Higher values indicate better intervention efficacy; PrBest: Probability of being the best intervention; MeanRank: Lower values indicate a higher ranking.
