# Supplementary material for: The impact of different types of exercise on sleep in sedentary populations: a systematic review and network meta-analysis
Source: PeerJ. 2026 Jun 9;14:e21037. doi: 10.7717/peerj.21037 (PMC13262546; doi:10.7717/peerj.21037)
Supplement: Supplemental Information 2 [file peerj-14-21037-s002.docx]

### Rationale and Contribution for the Systematic Review and Network Meta-Analysis

**1. Rationale**
In modern society, sedentary behavior has become increasingly prevalent due to technological advancements and lifestyle changes, significantly impacting sleep quality and overall health. Existing studies have shown that physical activity can improve sleep problems, particularly among sedentary individuals. Various forms of exercise—such as aerobic training, resistance training, tai chi, yoga, and Pilates—have demonstrated potential benefits for sleep; however, there is still uncertainty regarding which exercise modality is most effective in improving sleep quality for sedentary populations. While several studies have examined the impact of exercise on sleep, a comprehensive synthesis comparing the effectiveness of different exercise types is still lacking. Therefore, this systematic review and network meta-analysis aims to integrate high-quality randomized controlled trials (RCTs) to identify the most effective exercise modality for improving sleep outcomes in sedentary individuals.

**2. Contribution to Knowledge**
Previous meta-analyses have largely focused on specific exercise types (e.g., aerobic exercise or yoga) and their effects on sleep in general or clinical populations. To date, no study has systematically compared the relative effectiveness of multiple exercise modalities on sleep in sedentary individuals. This study is the first to use network meta-analysis to synthesize evidence from a broad range of recent RCTs, comparing aerobic exercise, resistance training, and mind-body interventions in this specific population. The key contributions and innovations of this study include:

1. A focused investigation on sedentary individuals and their specific sleep-related health concerns;
2. Inclusion of diverse exercise modalities and ranking of their effects through network meta-analysis;
3. Use of rigorous methodological standards, including PRISMA and the Cochrane Handbook guidelines, to ensure scientific quality;
4. Practical implications for clinical interventions, public health policymaking, and individual health management, making this work valuable for both theory and application.
